# Extending the Peptide/Protein Interaction Paradigm to a Protein/Protein Engagement Model in RiPP Biosynthesis

**DOI:** 10.1021/acschembio.5c00411

**Published:** 2025-08-08

**Authors:** Mujeeb A. Wakeel, Elizabeth A. Corbin, Andrew C. McShan, Vinayak Agarwal

**Affiliations:** † School of Chemistry and Biochemistry, 1372 Georgia Institute of Technology, Atlanta, Georgia 30332, United States; ‡ School of Biological Sciences, Georgia Institute of Technology, Atlanta, Georgia 30332, United States

## Abstract

Enzymatic post-translational modification of small precursor peptides generates a wide diversity of bioactive peptidic natural products. The interaction between the precursor peptide and the peptide modifying enzyme relies on recognition of the N-terminal region of the precursor peptide—termed the leader peptide—by the modifying enzyme. In this study, we describe a model for the recognition of atypically long and highly structured nitrile hydratase-like leader peptides (NHLPs) by an azoline forming YcaO cyclodehydratase. Predicated upon the unique structure of NHLPs, the binding model relies on protein/protein interactions between higher-order secondary and tertiary structures of the NHLP and the modifying enzyme. In light of previous findings, we report that different modifying enzymes bind to different molecular surfaces of the NHLPs. These findings illustrate the modularity of different NHLP structural features and how fine-tuning of intermolecular interactions is necessary for efficient catalysis.

## Graphical abstract

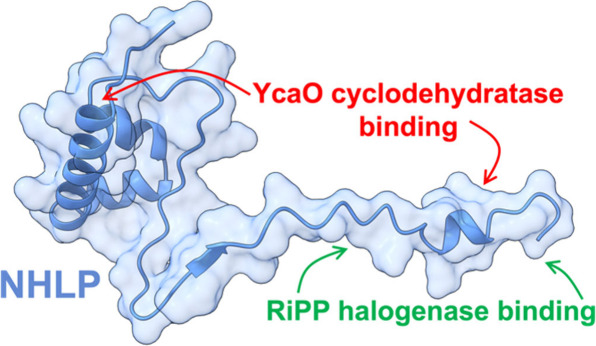

Post-translational modification of proteins during primary biological processes such as signaling and protein degradation relies on precise protein/protein interactions between modification enzymes and protein substrate(s). These interactions involve crosstalk between higher-order protein secondary and tertiary structural features that consist of amino acid residues that are spread across the entire protein sequence. The intricate nature of these interactions is richly illustrated by structural description of the protein/protein complexes involved in ubiquitination.^1,2^

Biomolecular interactions that underlie the biosynthesis of ribosomally synthesized and post-translationally modified peptides (RiPPs) adopt a different flavor. Here, a precursor peptide is recognized by precursor peptide-modifying RiPP biosynthetic enzymes. The RiPP precursor peptide is typically divided into an N-terminal leader region and a C-terminal core. The leader engages with the RiPP biosynthetic enzymes and directs the post-translational modification of the core.^3^ Typically, the leader peptides are short, bereft of tertiary structures—transiently stable solitary secondary structural elements may be present. Unlike the higher-order protein/protein interactions mentioned above, peptide/protein interactions involved in RiPP biosynthesis are predicated upon a short span of contiguous residues in leader peptides. Proteolytic removal of the modified core from the leader furnish the mature RiPP.

Among the RiPP biosynthetic enzymes are the ATP-dependent amide bond activating YcaO cyclodehydratases. Phosphorylation of the amide carbonyl manifests, among other modifications, as the dehydration of Cys, Ser, and Thr side chains to install azoline heterocycles in the core regions of RiPP precursor peptides (Figure 1A).^4–7^ Azolines, and their 2e^–^ oxidized counterparts—azoles—are present in numerous RiPPs such as the linear azol­(in)­e containing peptides, thiopeptides, and cyanobactins.^8–11^

**Figure 1:**
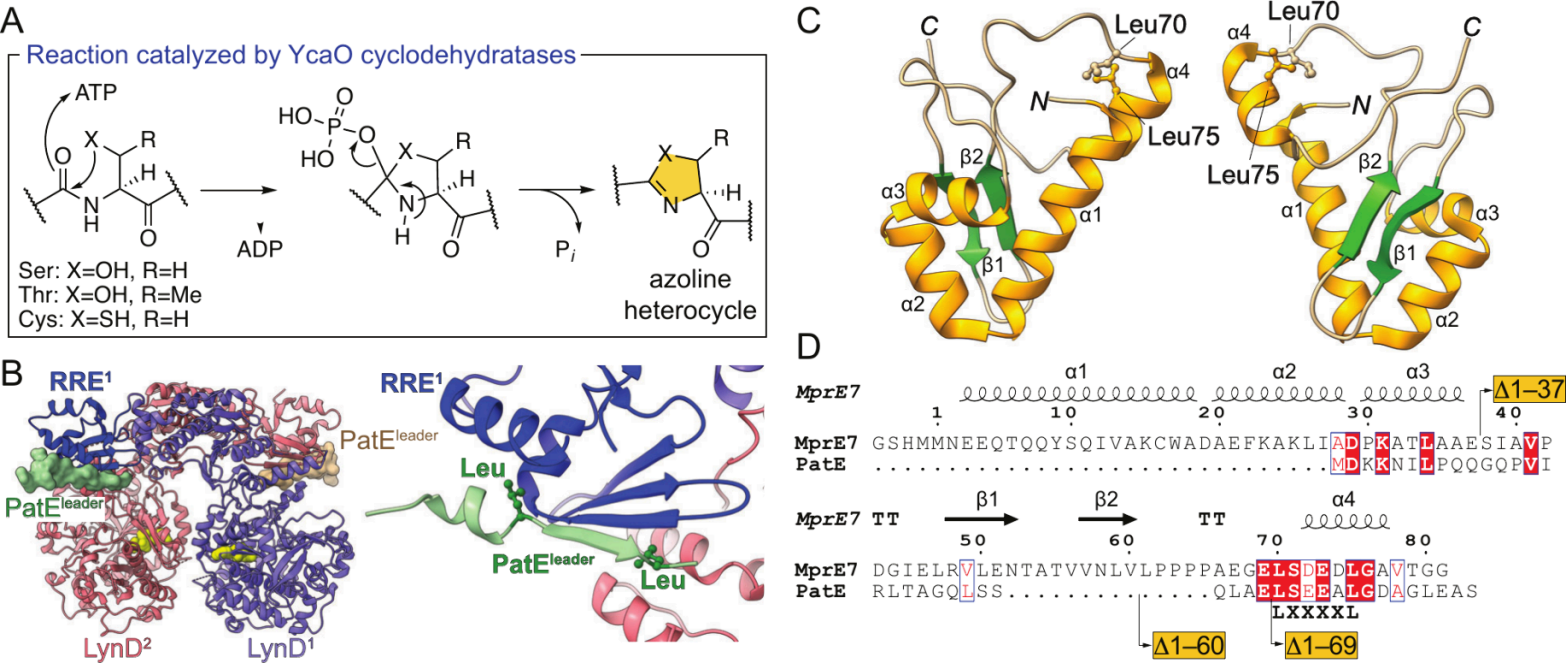
(A) Azoline synthesis catalyzed by YcaO cyclodehydratases. (B) Crystal structure of the PatE^leader^/LynD complex (PDB: 4V1T). ADP bound in the LynD active sites is shown as a yellow spheres. The PatE^leader^ peptides are shown in surface representation (left) with the RRE domain of one of the LynD monomers colored in a darker shade. The PatE^leader^ extends the RRE antiparallel β-sheet (right). Side chains of the Leu residues in the PatE^leader^ that constitute the L­(X)_4_L motif are shown in stick-ball representation. (C) Two views of the solution NMR structure of the MprE_7_ peptide (PDB: 8TB1). The N- and C-termini and secondary structural elements are labeled. The two Leu residues that constitute the L­(X)_4_L motif in the MprE_7_^leader^ are shown with the side chains in stick–ball representation. (D) Sequence alignment between MprE_7_^leader^ and PatE^leader^. The sites for the truncation of the MprE_7_ peptide are denoted (vide infra).

The leader region of the cyanobactin precursor peptide PatE was visualized bound to the YcaO cyclodehydratase LynD.^12–14^ The PatE^leader^/LynD crystal structure revealed the PatE^leader^ to be bound to the LynD RiPP recognition element (RRE) domain at the interface of two LynD monomers (Figure 1B).^15^ Prior studies had revealed the PatE^leader^ to be unstructured in aqueous buffers;^16^ however, upon binding to LynD, PatE^leader^ adopted a β-strand that extended the LynD RRE domain’s antiparallel β-sheet. This PatE^leader^ β-strand spanned the L­(X)_4_L motif; the two PatE^leader^ Leu residues constituting the L­(X)_4_L motif were verified to be important for PatE^leader^/LynD binding (Figure 1B).^13,14^

We have recently described the activity of the YcaO cyclodehydratase MprC that installs azoline heterocycles in the core regions of ten MprE_1_–MprE_10_ precursor peptides.^17^ Unlike the PatE^leader^ that is short (42 amino acids) and unstructured when *not* bound to a RiPP biosynthetic enzyme such as LynD, the MprE leaders are long (81 amino acids) and belong to the nitrile hydratase-like leader peptide (NHLP) family.^18,19^ We had previously shown that NHLPs such as the MprE_7_^leader^ possess a stable and rigid tertiary fold akin to a structured protein, and unlike the canonical short and unstructured RiPP leader peptides (Figure 1C).^20^ Thus, it was not clear if MprC would bind to the NHLP MprE_7_^leader^ in a manner analogous to the PatE^leader^/LynD interaction, or if new modalities would be adopted for the MprE_7_^leader^/MprC interaction. Note that the L­(X)_4_L motif is conserved in PatE^leader^ and MprE^leader^ (Figure 1D), as is the RRE domain in LynD and MprC (Figure 1B and vide infra).

MprC modified ten different substrate peptides, MprE_1_–MprE_10_, with the number of azoline rings installed in these peptides varying from two to six.^17^ To simplify the readout for MprC activity, we designed a core sequence containing a single Cys residue which would be modified by MprC to a thiazoline ring with an accompanying −18 Da mass loss; MprC prefers installing thiazole rings, as compared to (methyl)­oxazolines.^17^ The activity of MprC was combined with that of the flavin-dependent oxidase MprD which oxidizes the MprC-generated thiazoline to a thiazole resulting in a −20 Da mass loss as compared to the unmodified precursor peptide.^17^ A Pro residue was installed upstream of the Cys residue to provide a peptide fragmentation handle to localize the −20 Da mass loss to this Cys residue by liquid chromatography/mass spectrometry (LC/MS).^21^ The resulting -GGPACAAK core was appended to the MprE_7_^leader^; the NMR structure of the MprE_7_^leader^ has been determined.^20^ This chimeric substrate is henceforth termed the MprE_7_^leader^-GGPACAAK precursor peptide (Table S1). Upon in vitro treatment of MprE_7_^leader^-GGPACAAK with MprC and MprD, we observed product formation which was abolished in the absence of ATP/Mg^2+^ (Figures 2B–G). Identical modification of the precursor peptide was observed when the gene encoding MprE_7_^leader^-GGPACAAK peptide was coexpressed with *mprC* and *mprD* in *Escherichia coli* (Figure S1). These observations now set the stage with in vitro and in vivo assay procedures for interrogating MprE_7_^leader^/MprC binding with the extent of substrate conversion serving as a proxy for intermolecular interactions.

**Figure 2:**
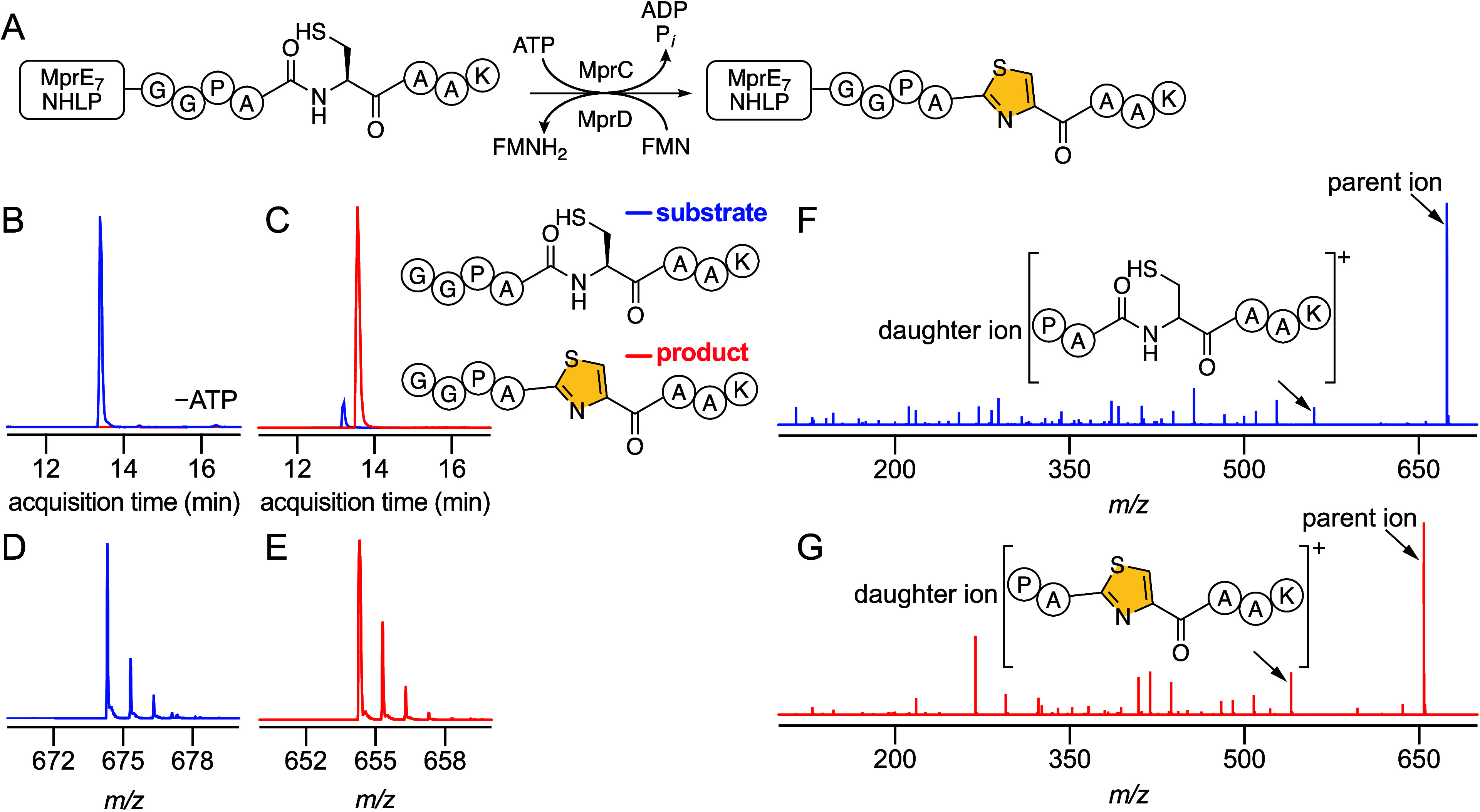
(A) Thiazole formation in the MprE_7_^leader^-GGPACAAK precursor peptide by MprC and MprD. MprD employs flavin mononucleotide (FMN) as a cofactor. Extracted ion chromatograms (EICs) for the [M + H]^+^ ions corresponding to the unmodified substrate core peptide (in blue) and the modified product core peptide (in red) observed in in vitro reactions with MprC/MprD (B) in the absence of ATP and (C) in the presence of ATP. MS^1^ isotopic distribution for (D) the unmodified core and (E) the modified core demonstrates the −20 Da mass loss corresponding to thiazole formation. MS^2^ fragmentation spectra for (F) the unmodified core and (G) the modified core with the parent ions and characteristic daughter ions labeled.

First, we evaluated the role of the two Leu residues in the L­(X)_4_L motif in mediating the MprE_7_^leader^/MprC interactions. As noted above, these Leu residues are conserved between the MprE_7_^leader^ and PatE^leader^ sequences and have been shown to mediate PatE^leader^/LynD interactions (Figure 1D).^13^ When these two Leu residues in MprE_7_^leader^-GGPACAAK—Leu70 and Leu75—were mutated to Ala, we observed loss of product formation in vivo; mutation of the Leu residues to Val and Phe rescued product formation (Table S1, Figure 3A, Figures S2–S5). Using circular dichroism (CD) spectroscopy and nano differential scanning fluorometry (nanoDSF), we verified that mutations at the L­(X)_4_L motif did not disrupt the structure and stability of the MprE_7_^leader^ (Figure 3B, Figures S6–S9). Taken together, these data imply that the reduction in substrate turnover upon L­(X)_4_L → A­(X)_4_A mutation was due to alterations in the MprE_7_^leader^/MprC interactions. This inference is consistent with the PatE^leader^/LynD binding model wherein the side chains of these two Leu residues in the PatE^leader^ make hydrophobic contacts with the LynD RRE domain.^13,14^ Thus, the binding of the NHLP (MprE_7_^leader^) to a YcaO cyclodehydratase (MprC) mirrors, at least in part, the binding of a shorter and unstructured leader peptide to the RRE domain.

**Figure 3:**
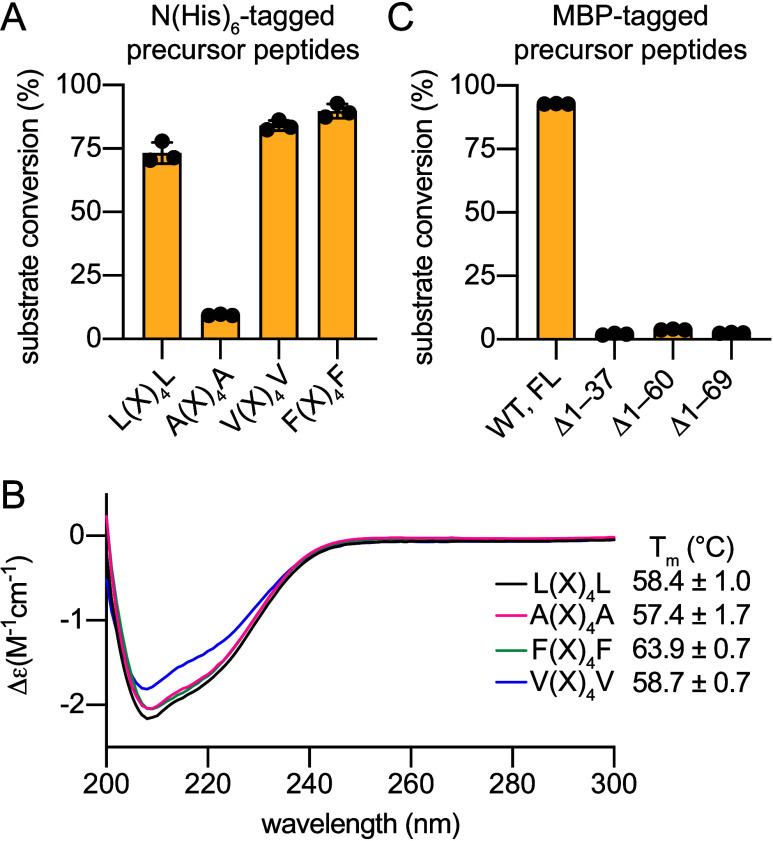
(A) Evaluation of MprC activity by monitoring substrate conversion when gene encoding the MprE_7_^leader^-GGPACAAK precursor peptide and mutants for the L­(X)_4_L motif was coexpressed in *E. coli* with *mprC* and *mprD*. The precursor peptides were expressed as fused to an N-terminal (His)_6_ tag. Substrate conversion was evaluated by LC/MS. (B) Far UV CD spectra for the wild-type MprE_7_^leader^ (L­(X)_4_L) and mutants thereof with the melting temperatures (*T*_m_) listed. (C) Evaluation of MprC activity when gene encoding the maltose binding protein-tagged (MBP-tagged) wild type MprE_7_^leader^-GGPACAAK peptide and truncation mutants thereof were coexpressed with *mprC* and *mprD* in *E. coli*. Data in panels (A)–(C) represent mean ± standard deviations from three independent experiments.

The L­(X)_4_L motif is in itself sufficient for mediating interactions between short RiPP precursor peptides and YcaO cyclodehydratases.^22^ To verify whether the L­(X)_4_L motif alone was also sufficient for NHLP engagement with YcaO cyclodehydratases, the MprE_7_^leader^-GGPACAAK peptide was truncated to eliminate the secondary structure elements from the NHLP while preserving the L­(X)_4_L motif (Table S1, Figure 1D). Three truncated variants were generated—(Δ1–37), (Δ1–60), and (Δ1–69)—and their in vivo conversion to products was evaluated relative to the full length MprE_7_^leader^-GGPACAAK peptide. All truncation variants demonstrated near complete loss of product formation (Figure 3C, Figures S10–S13). These data allow us to posit that while the hydrophobic Leu side chains in the L­(X)_4_L motif are indeed important for mediating MprE_7_^leader^/MprC interactions, the L­(X)_4_L motif is not the *only* determinant for NHLP engagement with the YcaO cyclodehydratase; it is likely that the NHLP secondary and tertiary structural features also participate in this interaction.

To further query the NHLP/YcaO interactions, we generated an AlphaFold 3 model of the 2:2 MprE_7_^leader^/MprC complex in the presence of ATP/Mg^2+^ (predicted template modeling (pTM) score = 0.83; interface predicted template modeling (ipTM) score = 0.79).^23^ The model was evaluated within the context of the available structures of the YcaO cyclodehydratases.^6,13,14,24^ AlphaFold 3 modeled the MprC dimer in excellent agreement with the experimental YcaO structures (Figure 4A). AlphaFold 3 also predicted the trihelical bundle at the N-terminus of the MprE_7_^leader^ correctly with the numerous hydrophobic side chains of residues from the NHLP α1−α3 helices burying a hydrophobic nucleus. Three salt bridge interactions observed in the solution NMR structure of MprE_7_^leader^ and which stabilize the NHLP tertiary structure were also maintained by AlphaFold 3 (Figure S14).^20^ The C-terminus of the MprE_7_^leader^ pointed toward the MprC active site where ATP/Mg^2+^ was modeled to be bound (Figure 4A).

**Figure 4:**
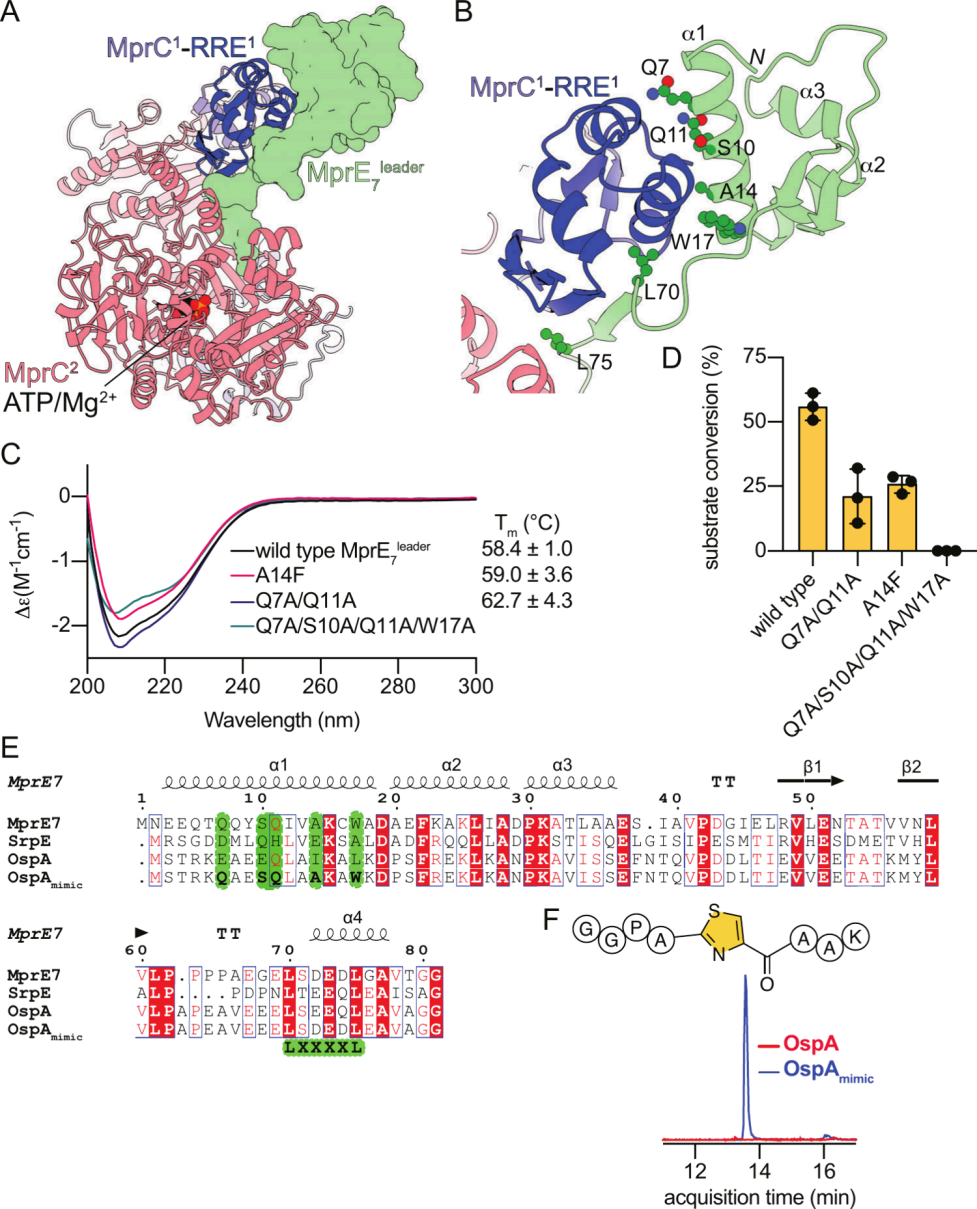
(A) Model of the MprE_7_^leader^/MprC complex. The MprE_7_^leader^ is shown in the surface representation colored green. (B) Zoomed in view of the MprE_7_^leader^ interaction with the MprC RRE domain. The side chains of key MprE_7_^leader^ residues discussed in the text are shown in a stick–ball representation. (C) CD spectra and *T*_m_ of the wild-type MprE_7_^leader^ peptide and mutant peptides discussed in the text. Since the Q7A/S10A/Q11A/W17A mutant peptide has no Trp residues, nanoDSF does not provide a reliable measurement of the *T*_m_. (D) Conversion of wild-type and mutant peptides to products monitored using in vitro enzymatic assays. Note that the conversion in in vitro reactions is typically lower that in in vivo co-expression assays that are denoted in Figure 3. (E) Sequence alignment between NHLPs MprE_7_^leader^, SrpE^leader^, and OspA^leader^. The residues in OspA^leader^ that were mutated to generate the OspA_mimic_^leader^ are highlighted in green. (F) EICs denoting the presence of the RiPP product when genes encoding the OspA^leader^-GGPACAAK (in red) and OspA_mimic_^leader^-GGPACAAK (in blue) were co-expressed with *mprC* and *mprD*.

The AlphaFold 3-predicted model demonstrated that the L­(X)_4_L motif in the MprE_7_^leader^ extended the antiparallel β-sheet of the MprC RRE domain with the Leu70 and Leu75 side chains making intimate contacts with the RRE domain (Figures 4A and 4B, Figure S15). Thus, the predicted model was in conformity with the biochemical data presented above and the previously described structure of the PatE^leader^/LynD complex. The MprE_7_^leader^ is bound by the RRE domain of one of the MprC monomers with the core peptide being delivered to the active site of the other MprC monomer (see Figure 4A, Figure S16).

The MprE_7_^leader^/MprC interaction model demonstrated extensive contact of one face of the MprE_7_^leader^ α1 helix with two helices of the MprC RRE domain (Figure 4B). This interaction was not observed in the PatE^leader^/LynD interaction model as the PatE^leader^ is short and does not possess analogous structural features. Side chains of residues that make up the MprE_7_^leader^ α1 helix—Gln7, Ser10, Gln11, Ala14, and Trp17—contact the MprC RRE domain. Among the principal interactions modeled by AlphaFold 3 were the hydrogen bonding contacts that side chain amides of the MprE_7_^leader^ Gln7 and Gln11 make with the MprC Asp50 and Try66 residues and burying the MprE_7_^leader^ Trp17 side chain indole into a hydrophobic cavity created by the MprC Ala63 and Tyr67 side chains.

To test the validity of the AlphaFold 3-predicted model, the MprE_7_^leader^ Gln7 and Gln11 residues were mutated to Ala. The MprE_7_^leader^-Q7A/Q11A demonstrated CD spectra and *T*_m_ similar to that of the wild-type MprE_7_^leader^ signifying no alteration of the NHLP structure and stability (see Figure 4C, as well as Figure S17). However, as compared to the wild-type substrate, the MprE_7_^leader^-Q7A/Q11A-GGPACAAK precursor peptide demonstrated a drastic reduction in thiazoline installation by MprC in vitro (see Figure 4D, Figure S18). When Gln7, Ser10, Gln11, and Trp17 were mutated together to Ala, we observed a complete loss in product formation, consistent with the proposed role of these residues in engaging with MprC (see Figure 4D, Figure S19).

We probed the interaction of the MprE_7_^leader^ α1 helix with the MprC RRE domain in another fashion. The Ala14 side chain methyl of the MprE_7_^leader^ was positioned proximal to the RRE helices; replacing Ala14 with a residue with a bulky side chain would then conceivably set up a steric clash with the RRE domain. Consistent with this hypothesis, when Ala14 was replaced with Phe, we observed a significant drop in the level of product formation with no alteration in the NHLP structure and stability (Figures 4C and D, Figures S20 and S21). These experimental findings were consistent with computational saturation mutagenesis of the MprE_7_^leader^ α1 helix residues Gln7, Ser10, Gln11, Ala14, Trp17 and the L­(X)_4_L motif residues Leu70 and Leu75 and evaluating the stability of the MprE_7_^leader^/MprC complexes thereof (Figure S22). Taken together, these data allow us to posit that the MprE_7_^leader^ α1 helix contacts the YcaO RRE domain and that these interacts are crucial—in addition to the L­(X)_4_L motif—in enabling the YcaO activity. Recently, the activity of the YcaO cyclodehydratase NstD has been described that installs azoline heterocycles in the NHLP precursor NstC; AlphaFold 3 predicts similar NstC^leader^/NstD binding interactions wherein the α1 helix of the NHLP is in intimate contact with the YcaO RRE and the NstC^leader^ L­(X)_4_L motif extends the NstD RRE β-sheet (Figure S23).^25^

We have previously experimentally shown that MprC is selective for the identity of the leader; NHLPs OspA^leader^ and SrpE^leader^ did not support MprC activity (Figures S24–S26).^17,26,27^ While OspA^leader^ and SrpE^leader^ possess the L­(X)_4_L motif, the residues that constitute the α1 helix are divergent (Figure 4E). Thus, we hypothesized that the MprC specificity could be dictated by the NHLP α1 helix. To test this hypothesis, residues of the OspA^leader^ were mutated to mimic the MprE_7_^leader^ α1 helix; these five residues at the 7, 10, 11, 14, and 17 positions were shown to be important in mediating the MprE_7_^leader^/MprC interaction (Figure 4D). Changes were also incorporated into the L­(X)_4_L motif to furnish the OspA_mimic_^leader^ sequence (Figure 4E). As compared to the OspA^leader^-GGPACAAK, which was not a viable substrate for MprC, the OspA_mimic_^leader^-GGPACAAK precursor peptide indeed demonstrated in vivo processing and product formation (see Figure 4F, as well as Figure S27). These data lend support to the bimodal interaction model of NHLP binding to the YcaO RRE domain such that the NHLP α1 helix and the L­(X)_4_L motif are both required for productive engagement. Interactions between a RiPP precursor peptide and the RiPP biosynthetic enzymes described here are not limited to the recognition of a few residues of a disordered leader peptide by partner enzyme; rather, these are protein/protein interactions involving higher-order tertiary structures from both the NHLP and the RRE domain. These findings bring forth the unique nature of NHLPs and how the NHLP structure participates in interaction with the RiPP biosynthetic enzymes.

The interaction model between the NHLP and the RRE domain described here is in contrast to the model that we had described for interaction of the NHLP SrpE^leader^ with the brominase SrpI.^20,28^ While the SrpE^leader^/SrpI interactions were dependent primarily on the Leu side chains that constituted the SrpE^leader^ L­(X)_4_L motif, SrpI—which lacks an RRE domain—made no contact with the structured NHLP nucleus. As such, all secondary structural elements of the NHLP were dispensable for SrpI binding. It is thus noteworthy that NHLPs possess multiple different modes of interacting with RiPP biosynthetic enzymes which would complicate NHLP leader peptide engineering for combinatorial RiPP production, as has been explored for other RiPP precursor peptides.^29,30^

## Supplementary Material



## Notes

Description of the experimental procedures, supplementary table detailing the peptide sequences used in this study, supplementary figures, and associated references (PDF)
