# Supplementary material for: Extending the Peptide/Protein Interaction Paradigm to a Protein/Protein Engagement Model in RiPP Biosynthesis
Source: ACS Chem Biol. 2025 Aug 8;20(9):2069–74. doi: 10.1021/acschembio.5c00411 (PMC12455567; doi:10.1021/acschembio.5c00411)
Supplement: Supplementary file 1 [file cb5c00411_si_001.pdf]

SUPPLEMENTARY INFORMATION FOR:

Extending the peptide/protein interaction paradigm to a protein/protein engagement model in RiPP biosynthesis

Mujeeb A. Wakeel,[1] Elizabeth A. Corbin,[1] Andrew C. McShan,[1] Vinayak Agarwal[1,2,]*

[1] School of Chemistry and Biochemistry, Georgia Institute of Technology, Atlanta, GA, 30332, USA

[2] School of Biological Sciences, Georgia Institute of Technology, Atlanta, GA, 30332, USA

* correspondence: vagarwal@gatech.edu

Supplementary Information document contains:

-- Supplementary Materials and Methods

-- Supplementary Table

-- Supplementary Figures

-- Supplementary References

SUPPLEMENTARY MATERIALS AND METHODS

Cloning and Gene Expression

A codon optimized gene encoding $MprE_7$ leader peptide was amplified from pET28$MprE_7$-TH1 using primers that appended the DNA sequence coding for the new core peptide GGPACAAK to the *mprE*$_7$ leader .[1] The resulting amplicon was subsequently cloned into pET-28a(+), generating pET28-$MprE_7$-GGPACAAK plasmid, which was subsequently used as template for generating the mutants used in this study. Plasmids were constructed using the Gibson assembly method in 10 µL reactions containing 50 ng linearized vector backbones, 150 ng insert DNA amplicons, and 5 µL Gibson Assembly Master Mix (New England Biolabs). Reactions were incubated at 50 °C for 50 min, followed by transformation into *Escherichia coli* DH5α competent cells. Negative selection pressure was applied using appropriate antibiotics and single colonies grown at 37 °C for 16 h were used for DNA extraction and sequencing. The plasmids pET28MBP-*mprC*, pET28MBP-*mprD*, and pCDFDuet-1-*mprC*-*mprD* were generated in a previous study and used as is for this study.[2]

General Protocol for Polymerase Chain Reaction (PCR)

Reactions were performed in 20 µL scale containing 0.15 ng/µL DNA template, 0.5 µM each forward and reverse primers, 1 µL dimethylsulfoxide (DMSO), and 10 µL PrimeSTAR Max Premix (2×) DNA polymerase (Takara Bio) using 3-step PCR protocol in 40 cycles. When necessary, PCR product mixtures were digested in 20 µL reactions containing 15 µL amplicon, 2 µL autoclaved Milli-Q H_2O, 2 µL 10× FastDigest buffer, and 1 µL FastDigest DpnI restriction enzyme (ThermoFisher Scientific) at 37 °C for 3 h, followed by cleaning and concentration using DNA Clean & Concentrator-5 kit (Zymo Research). DNA concentration was measured using NanoDrop One (Thermo Scientific).

Gene Expression and Protein Purification

Gene expression protocol:

Plasmid DNA was transformed in *E. coli* BL21(DE3) competent cells and grown on plates containing solid LB-agar media with appropriate antibiotics at 37 °C for 18 h. Colonies were used to inoculate 10 mL terrific broth (TB) media containing appropriate antibiotics and grown at 37 °C for 18 h, followed by transfer into fresh and autoclaved 1 L TB media supplemented with antibiotics. The 1 L TB cultures were incubated at 37 °C with shaking at 180 rpm with frequent measurement of OD_{600}. At OD_{600} 0.6–0.8, cultures were cooled to 18 °C before adding inducers (0.15 mM isopropyl-β-D-

thiogalactopyranoside (IPTG) for pET28(+) and pCDFDuet-1, 2 mg/mL L-(+)-arabinose for pGro7) to initiate the expression of proteins at 18 °C, 180 rpm for 18 h. GroEL and GroES chaperones (pGro7 — Takara) were co-expressed with MBP-MprC to ensure proper folding of MprC. Cultures were harvested by centrifugation (4,000×g, 25 min, 4 °C), followed by resuspension of cells in 40 mL lysis buffer (see below for buffer compositions) and subsequent cell rupture by sonication. Cell lysate was clarified by centrifugation (18,000×g, 45 min, 4 °C) to recover the supernatant containing soluble proteins.

Protein purification protocol using Ni-affinity:

5 mL His-Trap HP column charged with Ni^{2+} was equilibrated with 50 mL Milli-Q H_2O and 50 mL wash buffer before loading the supernatant onto the column at 2 mL/min flow rate. The loaded column was then washed extensively with wash buffer before eluting the protein of interest with elution buffer in a linear gradient from 0–100% elution buffer over 8 column volumes. Eluted fractions were analyzed with SDS-PAGE and fractions containing protein of interest were concentrated using Amicon centrifugal filters of appropriate molecular weight cutoffs (Millipore Sigma) and desalted into desalting buffer using PD-10 desalting columns (Cytiva), followed by protein concentration measurement by Bradford assay and storage at −80 °C in small aliquots. MBP-MprC was observed to lose activity after storage at −80 °C and was therefore used immediately in assays and not stored.

Buffer compositions:

Lysis buffer: 40 mM sodium phosphate (pH 7.5), 50 mM NaCl

Wash buffer: 40 mM sodium phosphate (pH 7.5), 50 mM NaCl, 30 mM imidazole

Elution buffer: 40 mM sodium phosphate (pH 7.5), 50 mM NaCl, 250 mM imidazole

Desalting buffer: 20 mM HEPES-Na (pH 7.5), 50 mM KCl, 10% v/v glycerol

Purification of peptides using size-exclusion chromatography:

Genes encoding the $MprE_7$-GGPACAAK, $MprE_7$-A(X_4)A-GGPACAAK mutant, $MprE_7$-F(X_4)F-GGPACAAK mutant, $MprE_7$-V(X_4)V-GGPACAAK mutant, $MprE_7$-A14F-GGPACAAK mutant, $MprE_7$-Q7A/Q11A-GGPACAAK mutant, and $MprE_7$-Q7A/S10A/Q11A/W17A-GGPACAAK mutant peptides were expressed in *E. coli* BL21(DE3) and purified on His-Trap HP column (Cytiva). To further purify these peptides to homogeneity as was required for circular dichroism (CD) and nano-differential scanning

fluorimetry (nanoDSF) experiments, a HiLoad 16/600 Superdex 75 pg column was equilibrated with 300 mL 1× Phosphate-Buffered Saline (PBS) buffer at 1.0 mL/min flow rate, followed by injection of 5 mL peptide sample onto the column. 200 mL 1× Phosphate-Buffered Saline (PBS) buffer (Fisher Scientific) was then passed through the column at 1.0 mL/min flow rate, collecting eluate in small fractions for analysis by SDS-PAGE.

Enzymatic Assays

In vivo:

Plasmids with the pET28(+) backbone and genes encoding the $MprE_7$-GGPACAAK, $MprE_7$-A(X_4)A-GGPACAAK mutant, $MprE_7$-F(X_4)F-GGPACAAK mutant, and $MprE_7$-V(X_4)V-GGPACAAK mutant were each co-expressed with pCDFDuet-1-*mprC*-*mprD*. Purified peptides were digested using GluC protease (Promega Corporation) in 200 µL reactions containing 1 µg/mL GluC, 100 µM purified peptide, 1 mM TCEP, and 10 mM HEPES-Na (pH 7.5), incubated at 30 °C for 3 h. GluC was used here because the two leucine residues that were mutated in the $MprE_7$ leader are potentially for LahT150 activity.[3] The pET28(+)-MBP plasmids harboring genes encoding the $MprE_7$-GGPACAAK, $MprE_7$(Δ1–37)-GGPACAAK, $MprE_7$(Δ1–60)-GGPACAAK, and $MprE_7$(Δ1–69)-GGPACAAK were each co-expressed with pCDFDuet-1-*mprC*-*mprD* followed by Ni-affinity purification and leader peptide excision using LahT150 protease in 200 µL reactions containing 20 µM LahT150, 100 µM purified peptide, 1 mM DTT, and 10 mM HEPES-Na (pH 7.5), incubated at 30 °C for 3 h. Digestion was quenched by addition of equal volume of MeOH containing 2% (v/v) formic acid (FA), and the resulting mixture centrifuged at 16,800×g for 30 min. Liquid chromatography/mass spectrometry (LC/MS) analysis was performed in triplicate for each peptide.

In vitro:

200 µL reactions containing 10 mM HEPES-Na (pH 7.5), 5 mM $MgCl_2$, 5 mM ATP, 5 mM TCEP, 100 µM flavin adenine mononucleotide (FMN), 15 µM MBP-MprC, 15 µM MBP-MprD, and 100 µM purified substrate peptide ($MprE_7$-GGPACAAK, $MprE_7$-A14F-GGPACAAK mutant, $MprE_7$-Q7A/Q11A-GGPACAAK mutant, or $MprE_7$-Q7A/S10A/Q11A/W17A-GGPACAAK mutant) were incubated at 30 °C for 16 h. The reaction mixtures were centrifuged to remove precipitates and then clear reaction mixtures were digested in reactions containing 20 µM LahT150, incubated at 30 °C for another 3 h. Equal volume of LC/MS grade methanol containing 2% (v/v) FA was added to the reaction mixtures and centrifuged at

16,800×g for 30 min prior to performing LC/MS analysis to quantify the MprC and MprD catalyzed thiazole formation. Reactions were performed in triplicate for each substrate peptide.

LC/MS Analysis

Clear samples obtained after the removal of the leader peptide by the appropriate protease were analyzed using an Agilent Poroshell 120 EC-C_{18} 2.7 μm 4.6×100 mm column on HPLC system coupled to an Agilent 6530 Q-TOF mass spectrometer. LC separation was carried out using 2% solvent B from 0–5 min, linear gradient to 100% solvent B from 5–18 min, 100% solvent B from 18–22 min, linear gradient to 2% solvent B from 22–24 min, and 2% solvent B from 24–30 min at 0.3 mL/min flow rate. MS data acquisition was performed in the positive ion mode using electrospray ionization (ESI) source and a MS range *m/z* 400–3000 Da. Auto MS/MS mode was enabled with collision induced dissociation (CID) energy of 30 V and MS/MS mass range was set to 50–3000 Da *m/z*. MS data analysis was performed using the Agilent MassHunter software. Extracted ion chromatograms (EICs) of unmodified substrate and modified product core peptides were obtained and the peak areas were calculated to estimate the percentage of thiazole formation in each enzymatic assay.

Solvent A: H_2O, 0.1% (v/v) formic acid

Solvent B: MeCN, 0.1% (v/v) formic acid

Circular Dichroism

Circular dichroism (CD) experiments were performed using an AP Chirascan-plus CD spectrometer using 300 μL of 50 μM purified peptides (unmodified $MprE_7$-GGPACAAK and mutants) in 1×PBS. CD data were acquired using a 1 mm pathlength cuvette (Starna Cell), monitoring profiles from 300 nm to 200 nm wavelength in 1 nm step at 20 °C. Three replicate datasets were collected for each sample, and the observed ellipticity (mdeg) was averaged and converted to molar ellipticity (Δε), which was then used to generate a CD spectrum for each peptide sample.

$$[\Delta\varepsilon] = \frac{M_{res} \times \theta_{obs}}{10 \times d \times C \times 3298}$$

where M_{res} (mean residue mass) = molecular weight/(n-1), n representing number of residues; θ_{obs} is the observed ellipticity in millidegrees; *C* is the concentration in mg/mL; and *d* is the pathlength in cm.

Nano Differential Scanning Fluorimetry

Nano differential scanning fluorimetry (NanoDSF) experiments were performed using a NanoTemper Prometheus NT.48 system. Approximately 5 µL of 50 µM purified peptides (unmodified $MprE_7$-GGPACAAK and mutants) in 1×PBS were loaded into Tycho NT.6 capillaries in triplicate, and fluorescence intensity was recorded at 350 nm and 330 nm to monitor the unfolding status of peptides as the temperature was increased from 20 °C to 95 °C in 1 °C/min thermal gradient. Melting temperatures (T_m) were determined from the inflection point of the first derivative curves obtained from the Prometheus Stability Analysis software.

AlphaFold 3 Modeling

The $MprE_7^{leader}$/MprC interaction model in 2:2 stoichiometry in the presence of two molecules of ATP and two Mg^{2+} ions was modeled by AlphaFold 3 using the online Alphafold server without using templates.

Supplementary Tables

Table S1: Peptide sequences used in this study

Enzyme/Peptide	Amino Acid Sequence
MBP	MGSSHHHHHH‡SSGLVPRGS†HMKIEEGKLVIWINGDKGYNGLAEVGK<br>KFEKDTGIKVTVEHPDKLEEKFPQVAATGDGPDIIFWAHDRFGGYAQSG<br>LLAEITPDKAFQDKLYPFTWDAVRYNGKLIAYPIAVEALSLIYNKDLLPN<br>PPKTWEEIPALDKELKAKGKSALMFNLQEPYFTWPLIAADGGYAFKYE<br>NGKYDIKDVGVDNAGAKAGLTFLVDLIKNKHMNADTDYSIAEAAFNK<br>GETAMTINGPWAWSNIDTSKVNYGVTVLPTFKGQPSKPFVGVLSAGIN<br>AASPNKELAKEFLENYLLTDEGLEAVNKDKPLGAVALKSYEEELAKDP<br>RIAATMENAQKGEIMPNIPQMSAFWYAVRTAVINAASGRQTVDEALKD<br>AQTNSSSHHHHHH‡ANSVPLVPRGS†ENLYFQS§GS
MprE$_7^{leader}$	MNEEQTQQYSQIVAKCWADAEFKAKLIADPKATLAAESIAVPDGIELRV<br>LENTATVVNLVLPPPPAEGELSDEDLGAVTGG
MprE$_7^{leader}$-<br>GGPACAAK	MNEEQTQQYSQIVAKCWADAEFKAKLIADPKATLAAESIAVPDGIELRV<br>LENTATVVNLVLPPPPAEGELSDEDLGAVTGGGGPACAAK
MprE$_7^{leader}$-A(X)$_4$A-<br>GGPACAAK	MNEEQTQQYSQIVAKCWADAEFKAKLIADPKATLAAESIAVPDGIELRV<br>LENTATVVNLVLPPPPAEGEASDEDAGAVTGGGGPACAAK
MprE$_7^{leader}$-F(X)$_4$F-<br>GGPACAAK	MNEEQTQQYSQIVAKCWADAEFKAKLIADPKATLAAESIAVPDGIELRV<br>LENTATVVNLVLPPPPAEGEFSDEDFGAVTGGGGPACAAK
MprE$_7^{leader}$-V(X)$_4$V-<br>GGPACAAK	MNEEQTQQYSQIVAKCWADAEFKAKLIADPKATLAAESIAVPDGIELRV<br>LENTATVVNLVLPPPPAEGEVSDEDVGAVTGGGGPACAAK
MBP-MprE$_7$(Δ1–37)-<br>GGPACAAK	MBP-<br>SIAVPDGIELRVLENTATVVNLVLPPPPAEGELSDEDLGAVTGGGGPACA<br>AK
MBP-MprE$_7$(Δ1–60)-<br>GGPACAAK	MBP-LPPPPAEGELSDEDLGAVTGGGGPACAAK
MBP-MprE$_7$(Δ1–69)-<br>GGPACAAK	MBP-LSDEDLGAVTGGGGPACAAK
MprE$_7^{leader}$-A14F-<br>GGPACAAK	MNEEQTQQYSQIVFKCWADAEFKAKLIADPKATLAAESIAVPDGIELRV<br>LENTATVVNLVLPPPPAEGELSDEDLGAVTGGGGPACAAK
MprE$_7^{leader}$-<br>Q7A/Q11A-<br>GGPACAAK	MNEEQTAQYSAIVAKCWADAEFKAKLIADPKATLAAESIAVPDGIELRV<br>LENTATVVNLVLPPPPAEGELSDEDLGAVTGGGGPACAAK
MprE$_7^{leader}$-<br>Q7A/S10A/Q11A/W<br>17A-GGPACAAK	MNEEQTAQYAAIVAKCAADAEFKAKLIADPKATLAAESIAVPDGIELRV<br>LENTATVVNLVLPPPPAEGELSDEDLGAVTGGGGPACAAK
OspA^{leader}-<br>GGPACAAK	MSTRKEAEEQLAIKALKDPSFREKLKANPKAVISSEFNTQVPDDLTIEV<br>VEETATKMYLVLPAPEAVEEELSEEQLEAVAGGGGPACAAK
OspA$_{mimic}^{leader}$-<br>GGPACAAK	MSTRKQAESQLAAKAWKDPSFREKLKANPKAVISSEFNTQVPDDLTIE<br>VVEETATKMYLVLPAPEAVEEELSDEDLEAVAGGGGPACAAK

‡His$_6$ tag, †thrombin cleavage site, §TEV protease cleavage site

Supplementary Figures

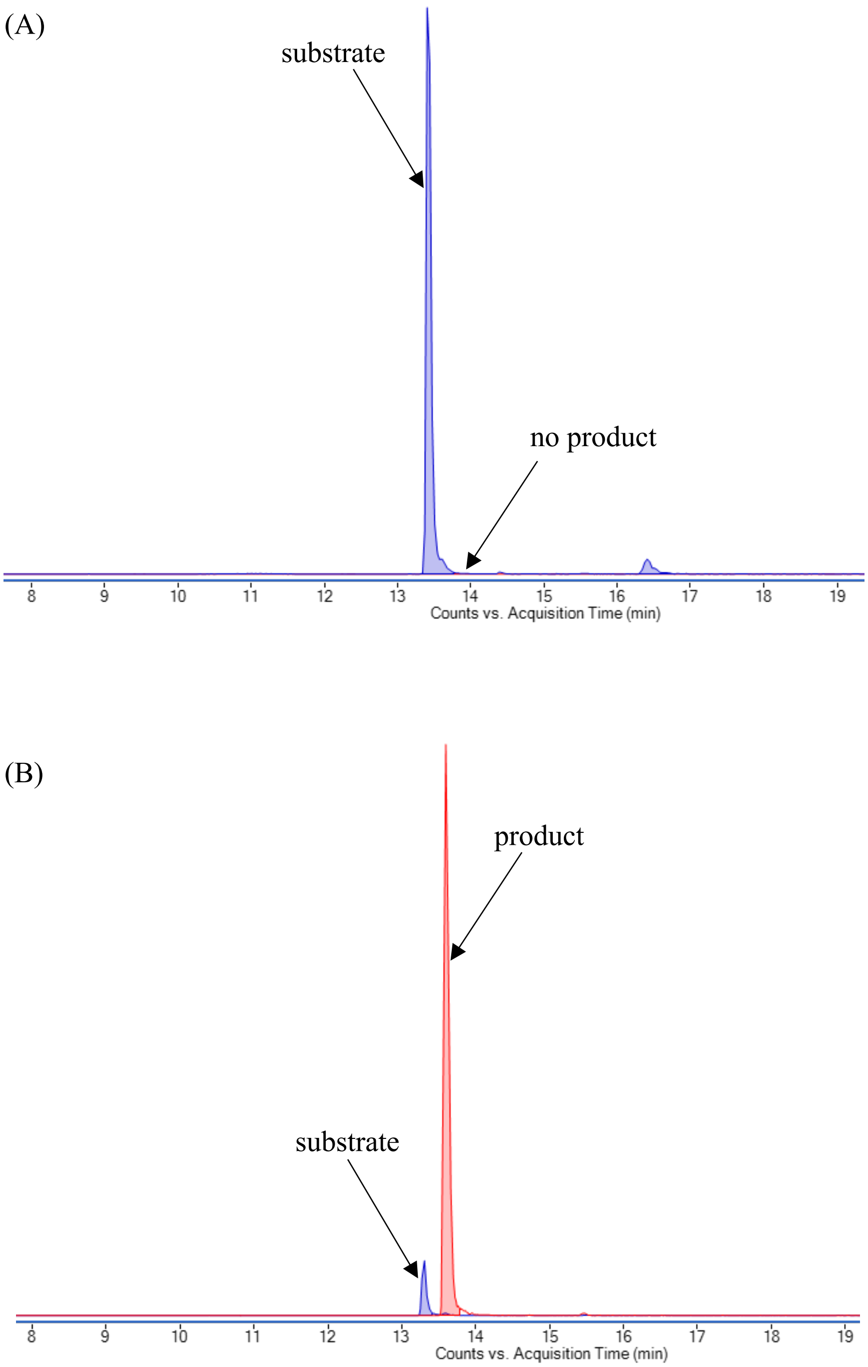

Figure S1: Extracted ion chromatograms (EICs) for the $[M+H]^+$ ions corresponding to the unmodified substrate core peptide (in blue) and the modified product core peptide (in red) observed when the gene encoding MprE$_7^{leader}$-GGPACAAK peptide was (A) expressed without *mprC* and *mprD* and (B) co-expressed with *mprC* and *mprD* in *E. coli.* Core peptides were excised by digestion by LahT150.

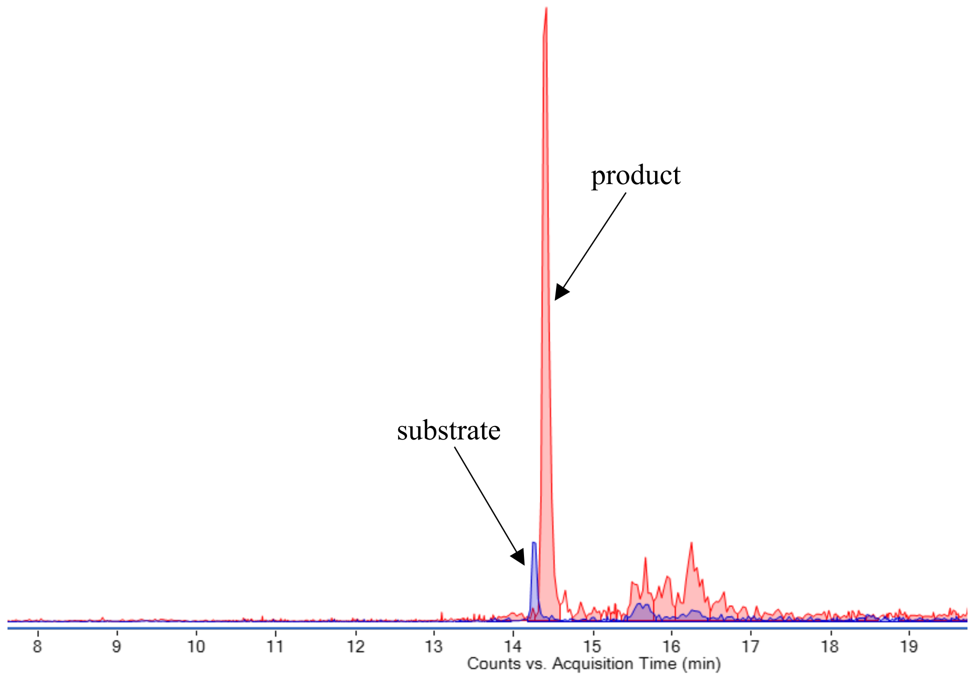

Figure S2: EICs for the $[M+H]^+$ ions corresponding to the unmodified substrate core peptide (in blue) and the modified product core peptide (in red) observed when the gene encoding $MprE_7^{leader}$-GGPACAAK peptide was co-expressed with *mprC* and *mprD* in *E. coli.*

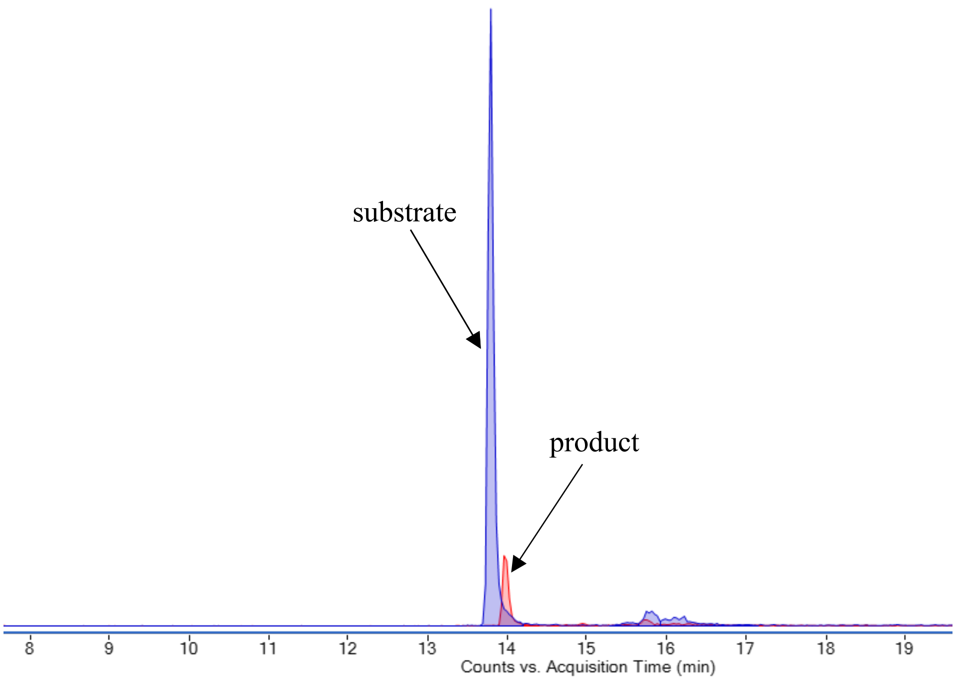

Figure S3: EICs for the $[M+H]^+$ ions corresponding to the unmodified substrate core peptide (in blue) and the modified product core peptide (in red) observed when the gene encoding $MprE_7^{leader}$-A(X_4)A-GGPACAAK peptide was co-expressed with *mprC* and *mprD* in *E. coli.*

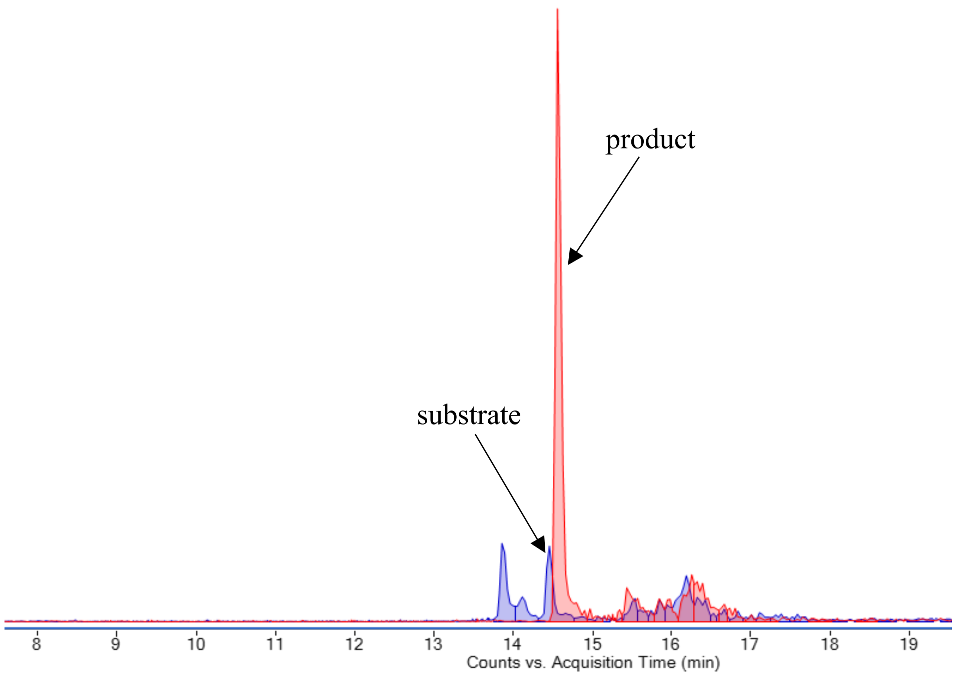

Figure S4: EICs for the $[M+H]^+$ ions corresponding to the unmodified substrate core peptide (in blue) and the modified product core peptide (in red) observed when the gene encoding $MprE_7^{leader}$-F(X_4)F-GGPACAAK peptide was co-expressed with *mprC* and *mprD* in *E. coli.*

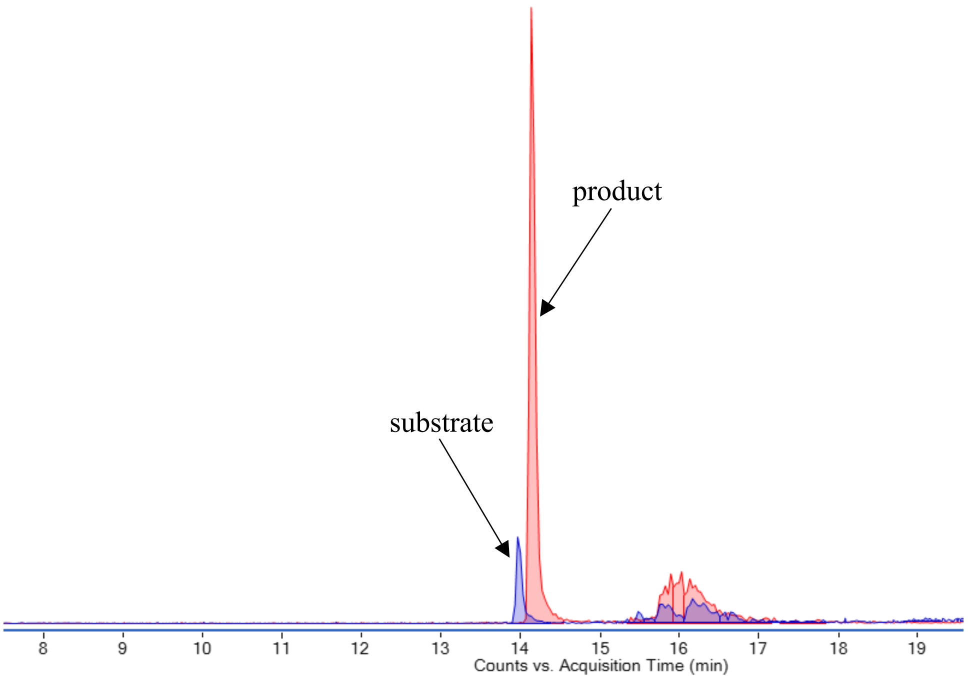

Figure S5: EICs for the $[M+H]^+$ ions corresponding to the unmodified substrate core peptide (in blue) and the modified product core peptide (in red) observed when the gene encoding $MprE_7^{leader}$-V(X_4)V-GGPACAAK peptide was co-expressed with *mprC* and *mprD* in *E. coli.*

(A)

(B)

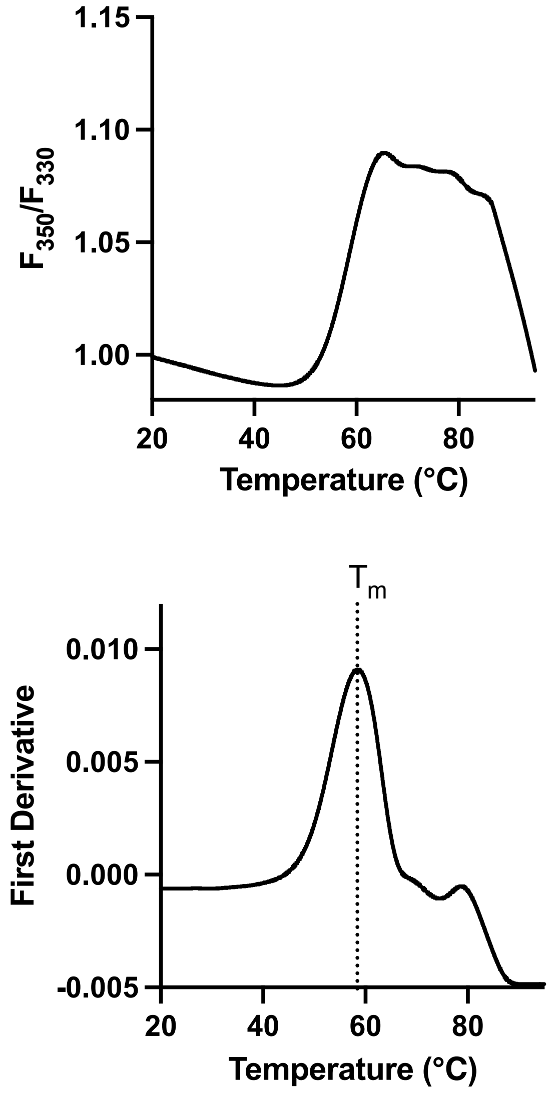

Figure S6: Thermal unfolding of $MprE_7^{leader}$-GGPACAAK peptide. (A) Plot of F_{350}/F_{330} fluorescence ratio against temperature, showing the thermal denaturation of $MprE_7^{leader}$-GGPACAAK peptide. (B) First derivative of the F_{350}/F_{330} plot to determine the melting point (T_m) of $MprE_7^{leader}$-GGPACAAK peptide.

(A)

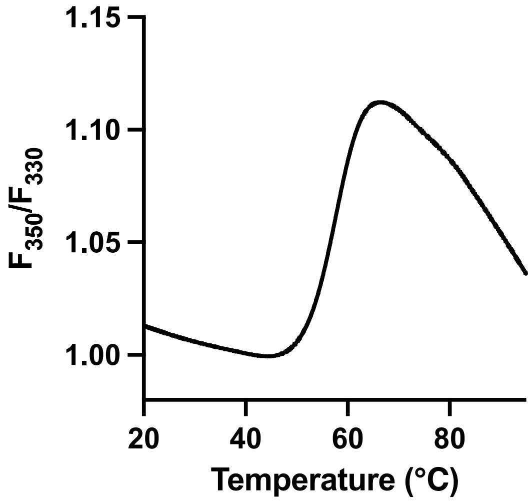

(B)

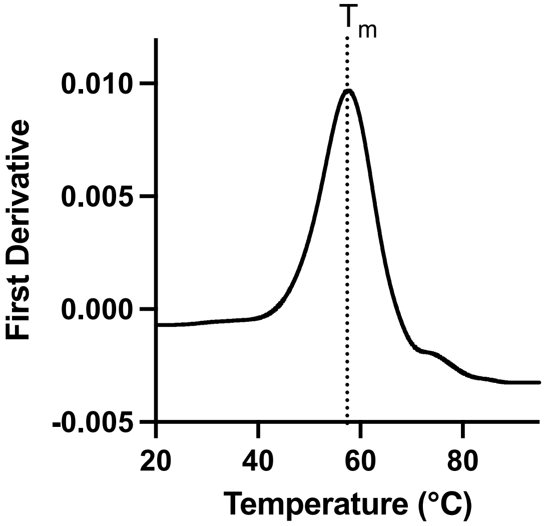

Figure S7: Thermal unfolding of $MprE_7^{leader}$-A(X_4)A-GGPACAAK peptide. (A) Plot of F_{350}/F_{330} fluorescence ratio against temperature, showing the thermal denaturation of $MprE_7^{leader}$-A(X_4)A-GGPACAAK peptide. (B) First derivative of the F_{350}/F_{330} plot to determine the melting point (T_m) of $MprE_7^{leader}$-A(X_4)A-GGPACAAK peptide.

(A)

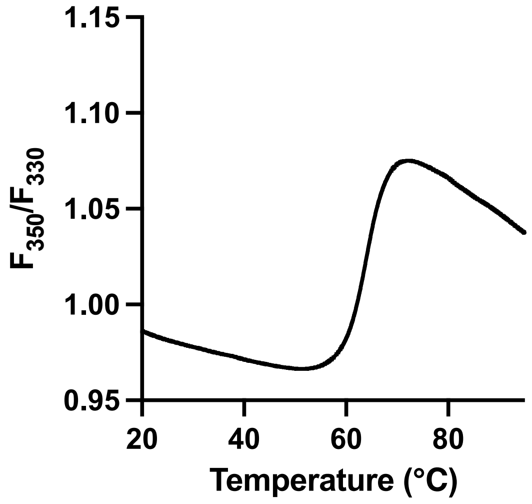

(B)

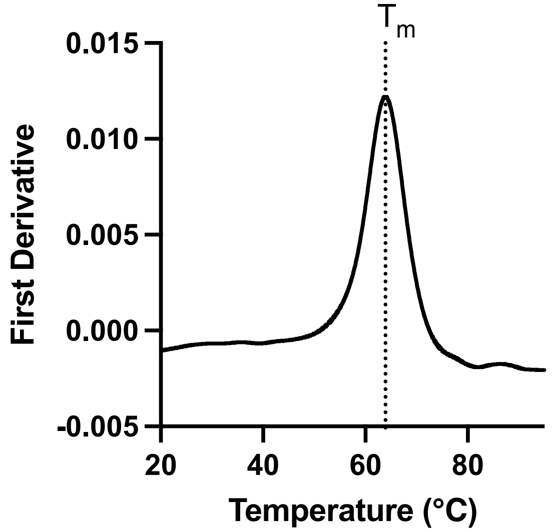

Figure S8: Thermal unfolding of MprE$_7^{leader}$-F(X$_4$)F-GGPACAAK peptide. (A) Plot of F_{350}/F_{330} fluorescence ratio against temperature, showing the thermal denaturation of MprE$_7^{leader}$-F(X$_4$)F-GGPACAAK peptide. (B) First derivative of the F_{350}/F_{330} plot to determine the melting point (T_m) of MprE$_7^{leader}$-F(X$_4$)F-GGPACAAK peptide.

(A)

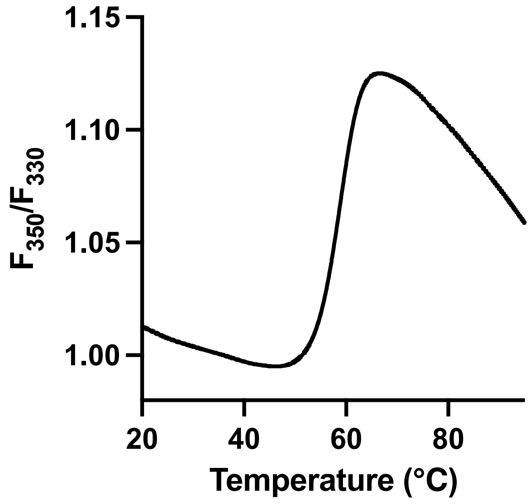

(B)

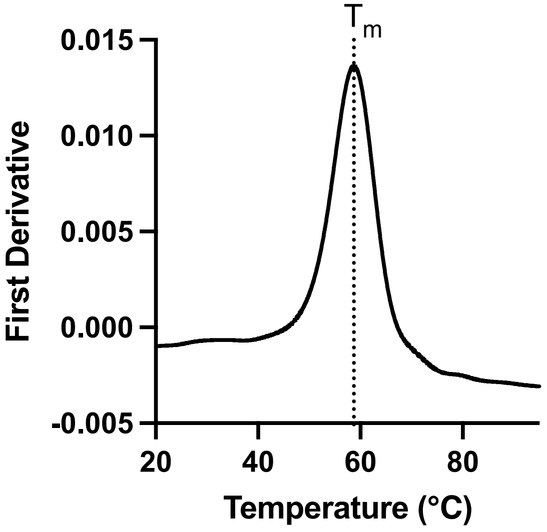

Figure S9: Thermal unfolding of $MprE_7^{leader}$-V(X_4)V-GGPACAAK peptide. (A) Plot of F_{350}/F_{330} fluorescence ratio against temperature, showing the thermal denaturation of $MprE_7^{leader}$-V(X_4)V-GGPACAAK peptide. (B) First derivative of the F_{350}/F_{330} plot to determine the melting point (T_m) of $MprE_7^{leader}$-V(X_4)V-GGPACAAK peptide.

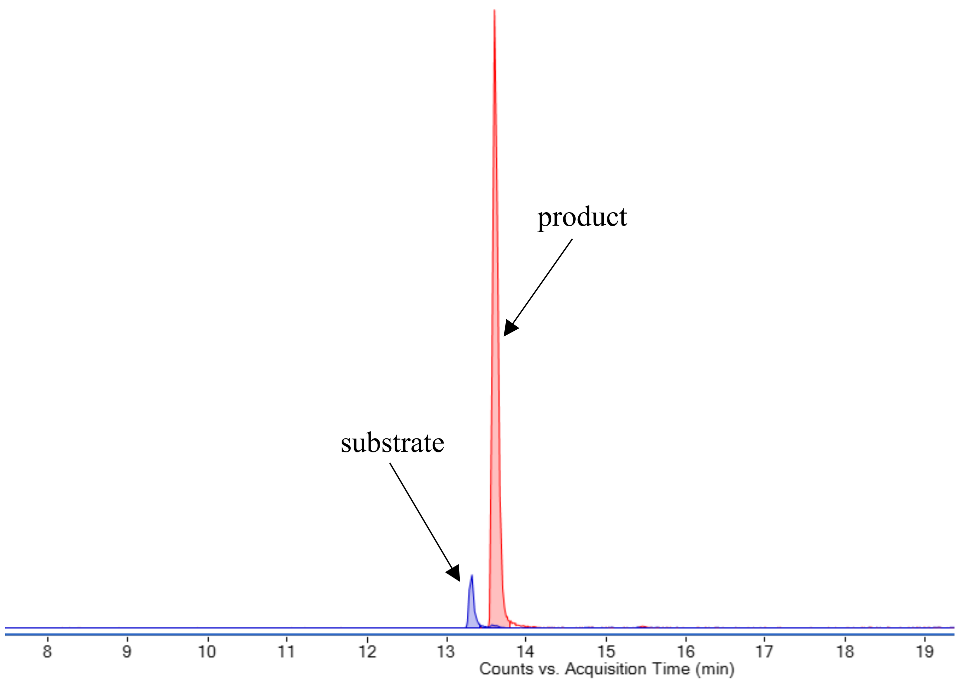

Figure S10: EICs for the $[M+H]^+$ ions corresponding to the unmodified substrate core peptide (in blue) and the modified product core peptide (in red) observed when the gene encoding MBP-$MprE_7^{leader}$-GGPACAAK peptide was co-expressed with *mprC* and *mprD* in *E. coli.* Because the NHLP truncation compromises solubility of the precursor peptides, the wild type and the truncated peptides were expressed as MBP-fusion proteins and not N-His_6 tagged peptides.

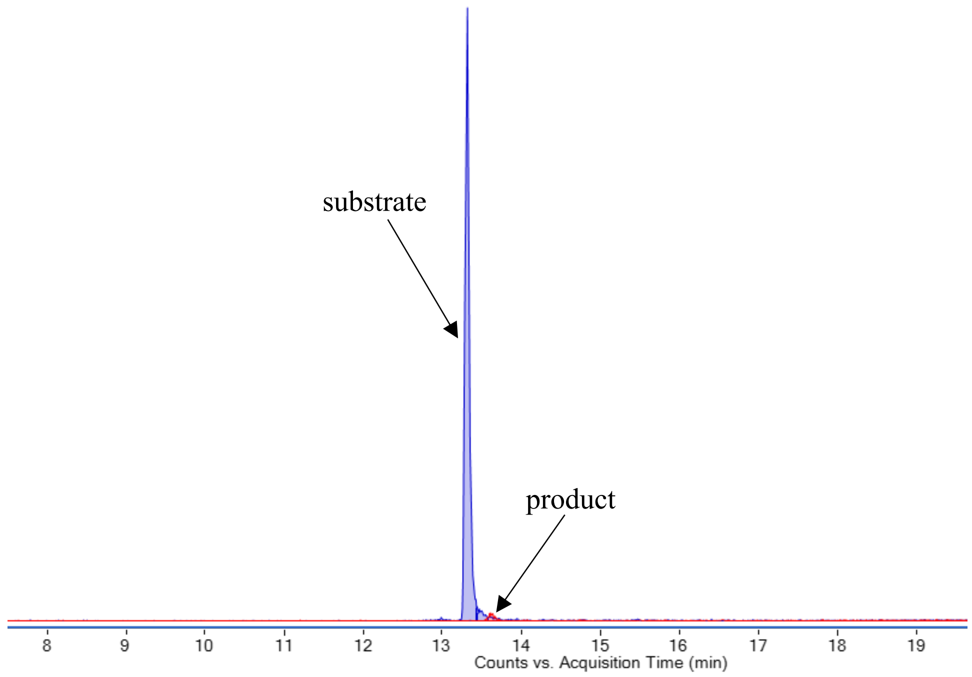

Figure S11: EICs for the $[M+H]^+$ ions corresponding to the unmodified substrate core peptide (in blue) and the modified product core peptide (in red) observed when the gene encoding MBP-MprE$_7$(Δ1–37)-GGPACAAK peptide was co-expressed with *mprC* and *mprD* in *E. coli.*

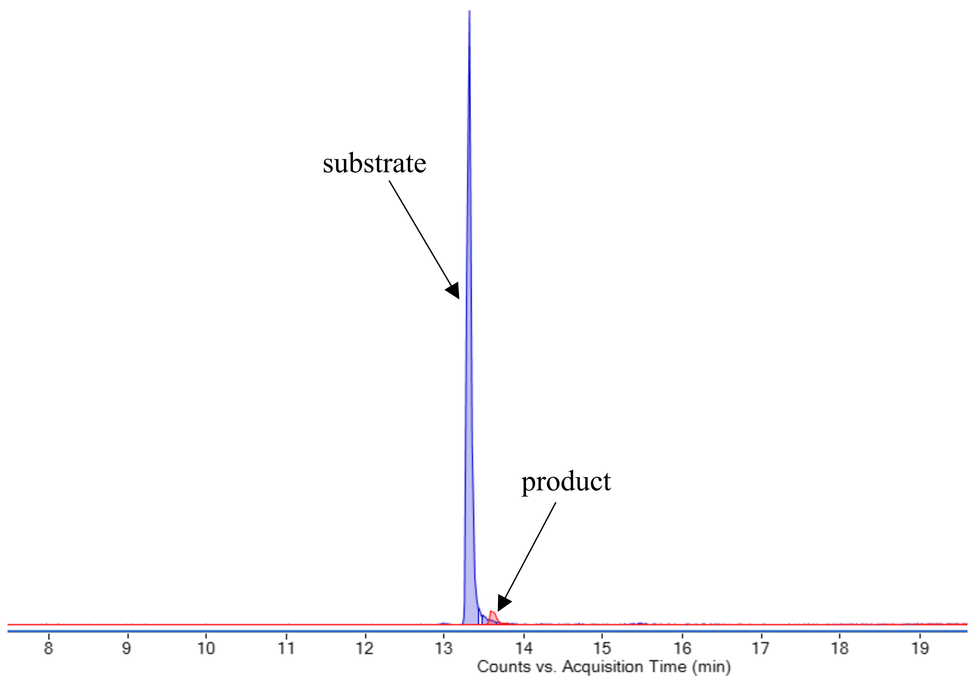

Figure S12: EICs for the $[M+H]^+$ ions corresponding to the unmodified substrate core peptide (in blue) and the modified product core peptide (in red) observed when the gene encoding MBP-MprE$_7$(Δ1–60)-GGPACAAK peptide was co-expressed with *mprC* and *mprD* in *E. coli.*

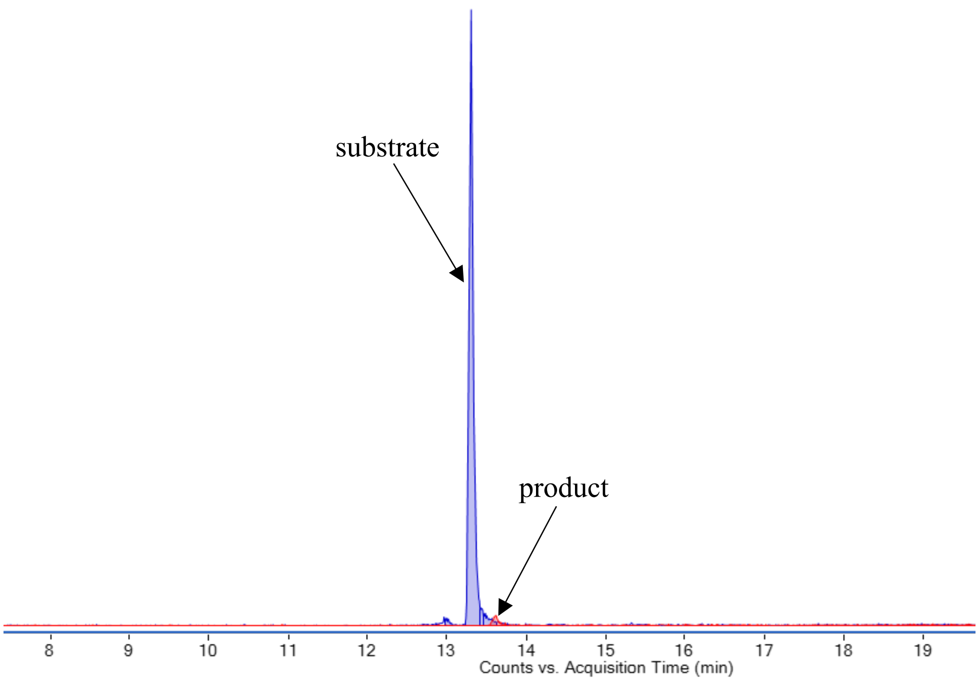

Figure S13: EICs for the $[M+H]^+$ ions corresponding to the unmodified substrate core peptide (in blue) and the modified product core peptide (in red) observed when the gene encoding MBP-MprE$_7$(Δ1–69)-GGPACAAK peptide was co-expressed with *mprC* and *mprD* in *E. coli.*

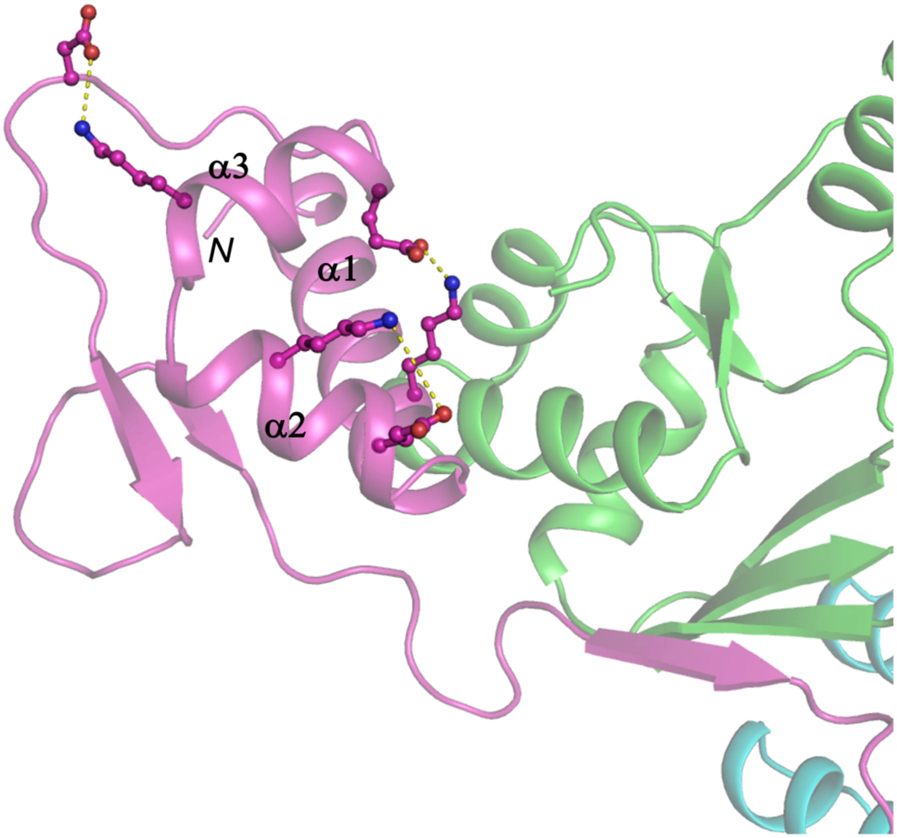

Figure S14: Cartoon representation of the MprE$_7$^{leader} (in pink) bound to the RRE domain of MprC (in green) with side chains involved in salt bridge interactions shown in stick-ball representation. The three helices characteristic of the NHLP structure are labeled. The representation is derived from the AlphaFold 3 model of the MprE$_7$^{leader}/MprC complex.

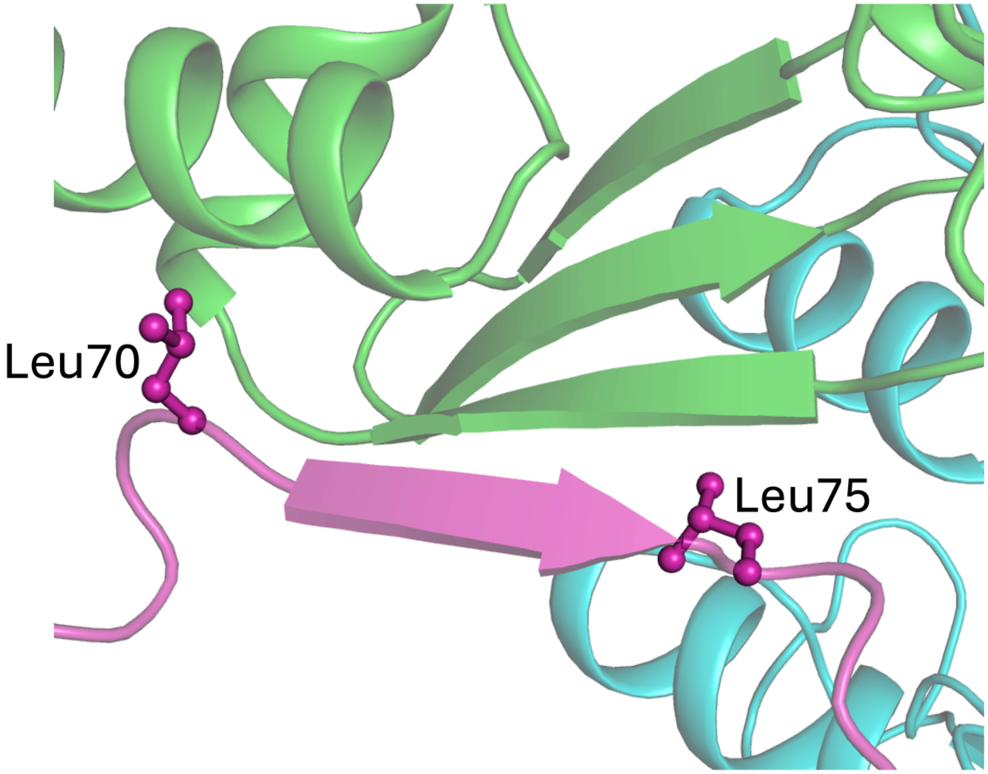

Figure S15: The MprE$_7^{leader}$ (in pink) extends the three stranded anti-parallel β-sheet of the MprC RRE domain (in green) with an additional antiparallel β-strand. This region of the MprE$_7^{leader}$ spans the L(X)$_4$L motif and the two Leu side chains are shown in stick-ball representation. The other MprC monomer is shown in cyan. The representation is derived from the AlphaFold 3 model of the MprE$_7^{leader}$/MprC complex.

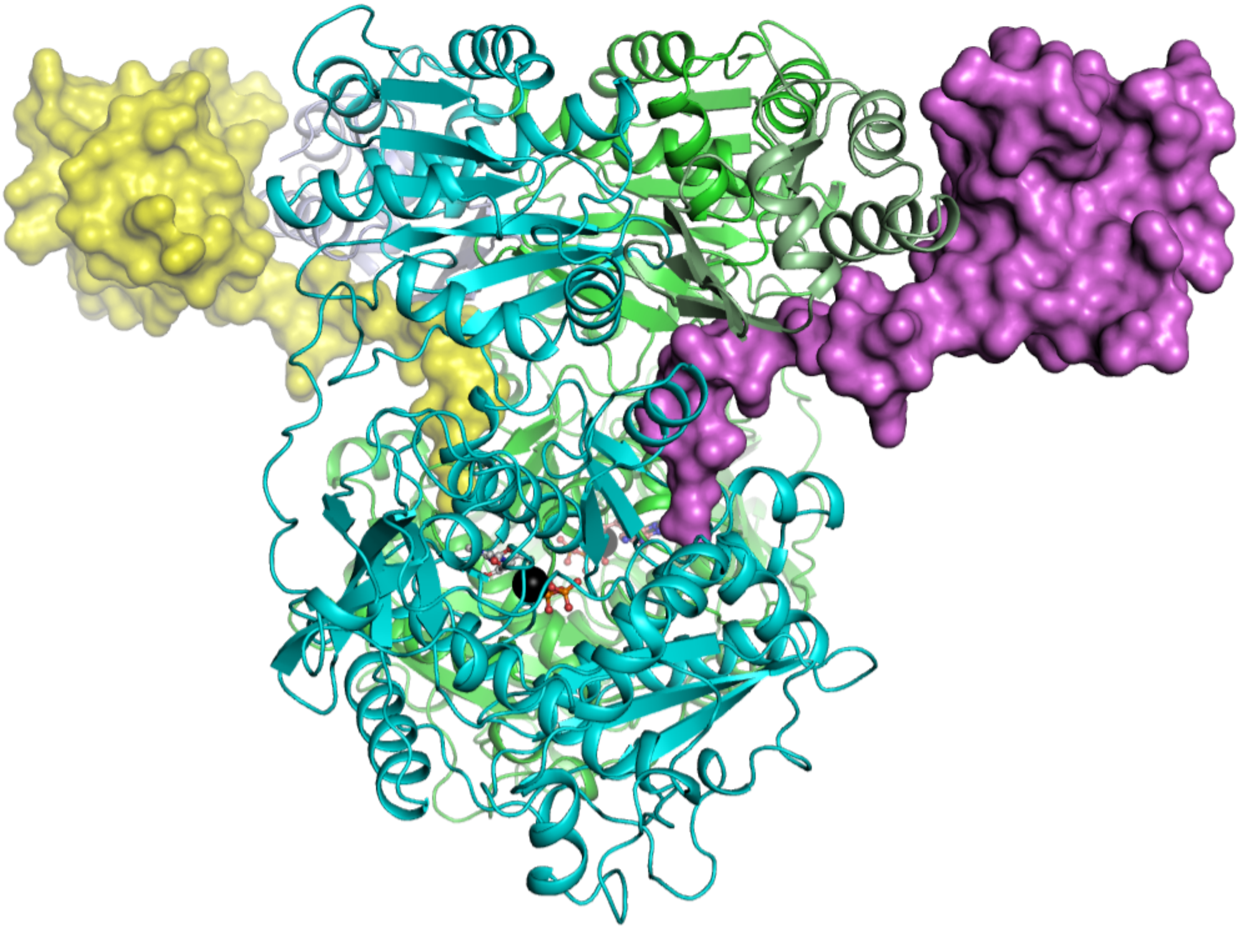

Figure S16: The $MprE_7^{leader}$ (in pink, surface representation) is bound to the RRE domain of the MprC monomer that is colored green (cartoon representation) while delivering the core to the active site of the other MprC monomer that is colored cyan (cartoon representation). Note that the MprC active site is denoted by ATP (stick-ball representation) and the bound Mg^{2+} ion (black sphere). The representation is derived from the AlphaFold 3 model of the $MprE_7^{leader}$/MprC complex.

(A)

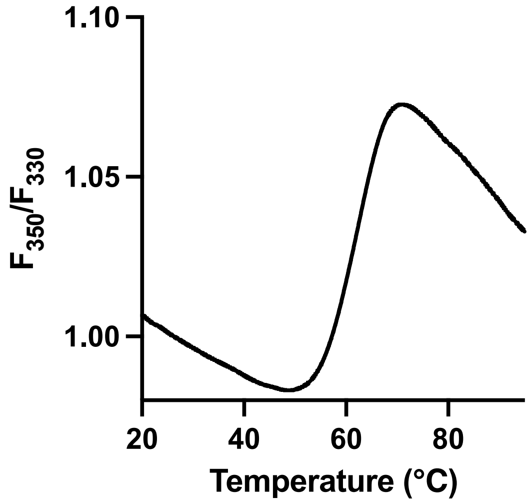

(B)

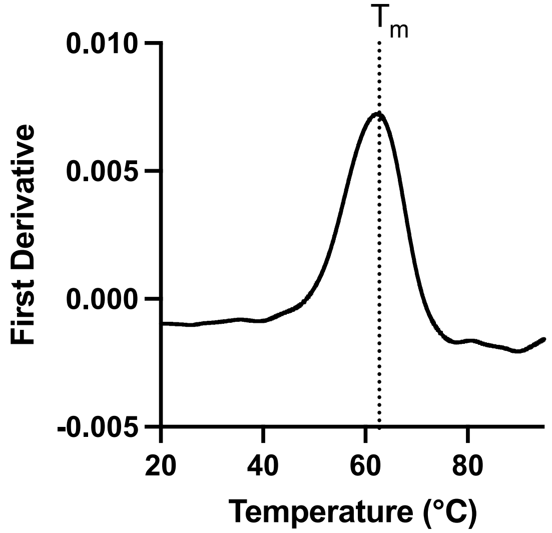

Figure S17: Thermal unfolding of $MprE_7^{leader}$-Q7A/Q11A-GGPACAAK peptide. (A) Plot of F_{350}/F_{330} fluorescence ratio against temperature, showing the thermal denaturation of $MprE_7^{leader}$-Q7A/Q11A-GGPACAAK peptide. (B) First derivative of the F_{350}/F_{330} plot to determine the melting point (T_m) of $MprE_7^{leader}$-Q7A/Q11A-GGPACAAK peptide.

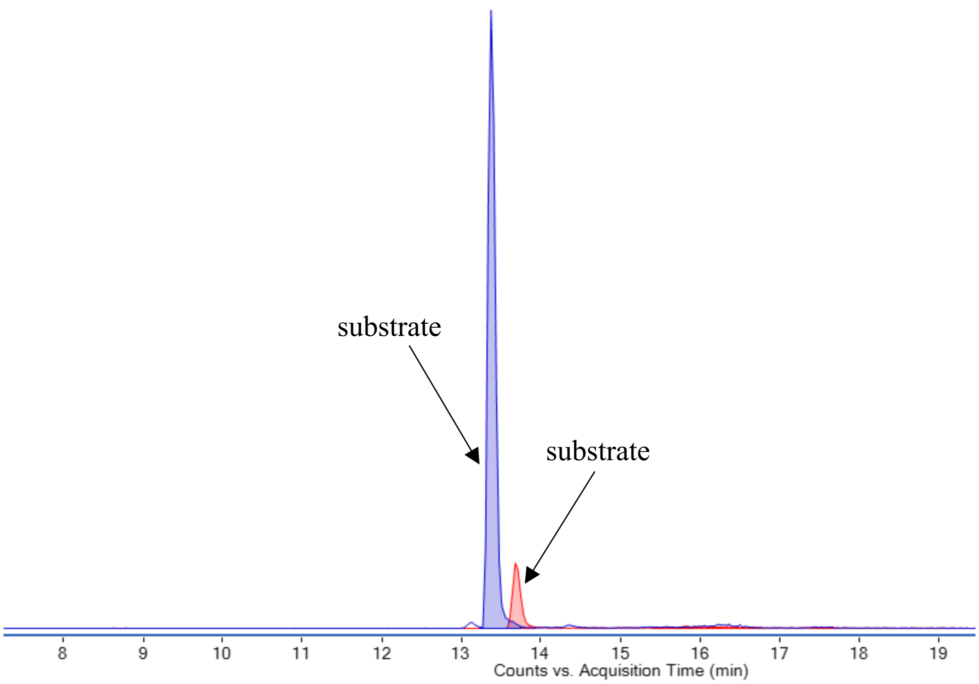

Figure S18: EICs for the $[M+H]^+$ ions corresponding to the unmodified substrate core peptide (in blue) and the modified product core peptide (in red) observed when purified $MprE_7^{leader}$-Q7A/Q11A-GGPACAAK peptide was incubated with MprC and MprD enzymes.

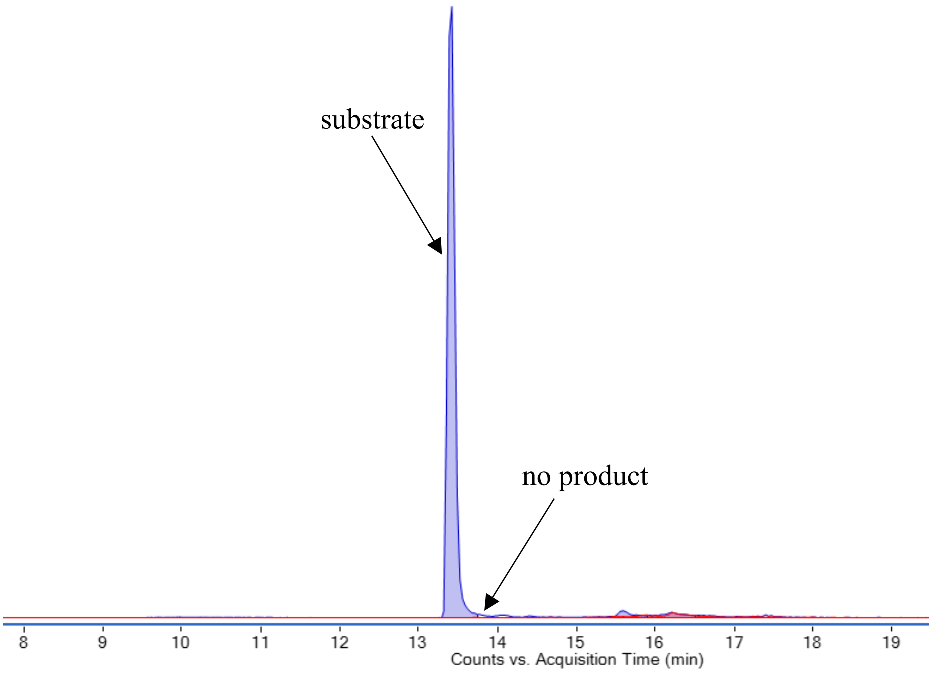

Figure S19: EICs for the $[M+H]^+$ ions corresponding to the unmodified substrate core peptide (in blue) and the modified product core peptide (in red) observed when purified $MprE_7^{leader}$-Q7A/S10A/Q11A/W17A-GGPACAAK peptide was incubated with MprC and MprD enzymes.

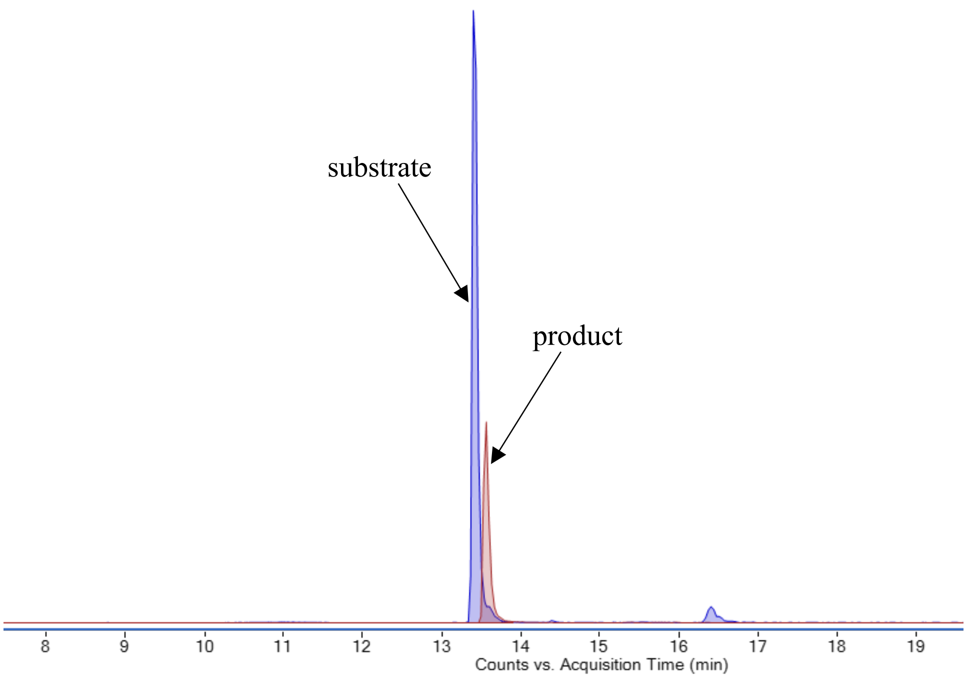

Figure S20: EICs for the $[M+H]^+$ ions corresponding to the unmodified substrate core peptide (in blue) and the modified product core peptide (in red) observed when purified $MprE_7^{leader}$-A14F-GGPACAAK peptide was incubated with MprC and MprD enzymes.

(A)

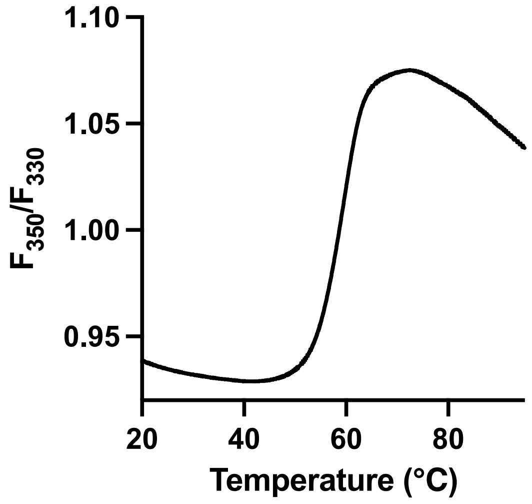

(B)

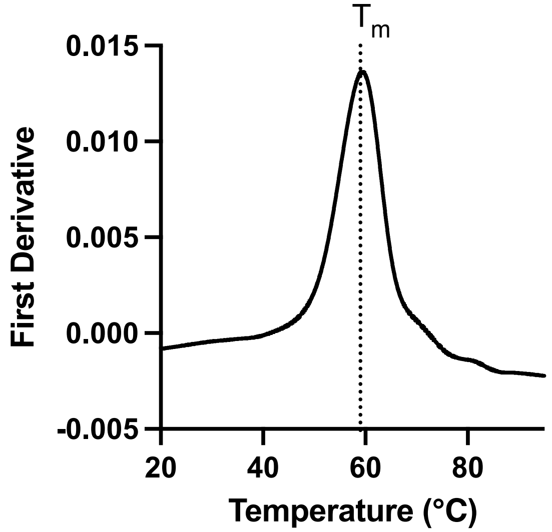

Figure S21: Thermal unfolding of MprE$_7$^{leader}-A14F-GGPACAAK peptide. (A) Plot of F_{350}/F_{330} fluorescence ratio against temperature, showing the thermal denaturation of MprE$_7$^{leader}-A14F-GGPACAAK peptide. (B) First derivative of the F_{350}/F_{330} plot to determine the melting point (T_m) of MprE$_7$^{leader}-A14F-GGPACAAK peptide.

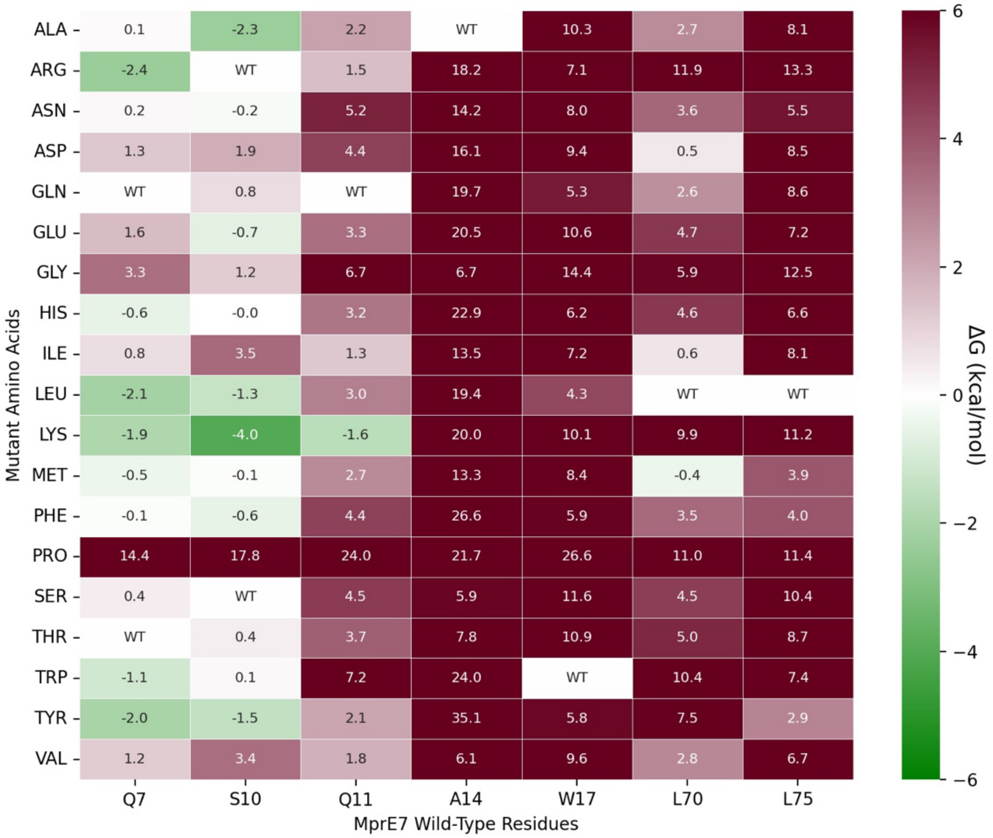

Figure S22: Heatmap of *in silico* site saturation mutagenesis of $MprE_7^{leader}$ that contact MprC in the $MprE_7^{leader}$/MprC complex derived from the AlphaFold 3. The mutant structures were modeled and scored within Rosetta Scripts. Green indicates the mutation made the total energy of the $MprE_7^{leader}$/MprC complex more stable, while red indicates the mutation made the total energy of the $MprE_7^{leader}$/MprC complex less stable. WT indicates "wild-type" residue.

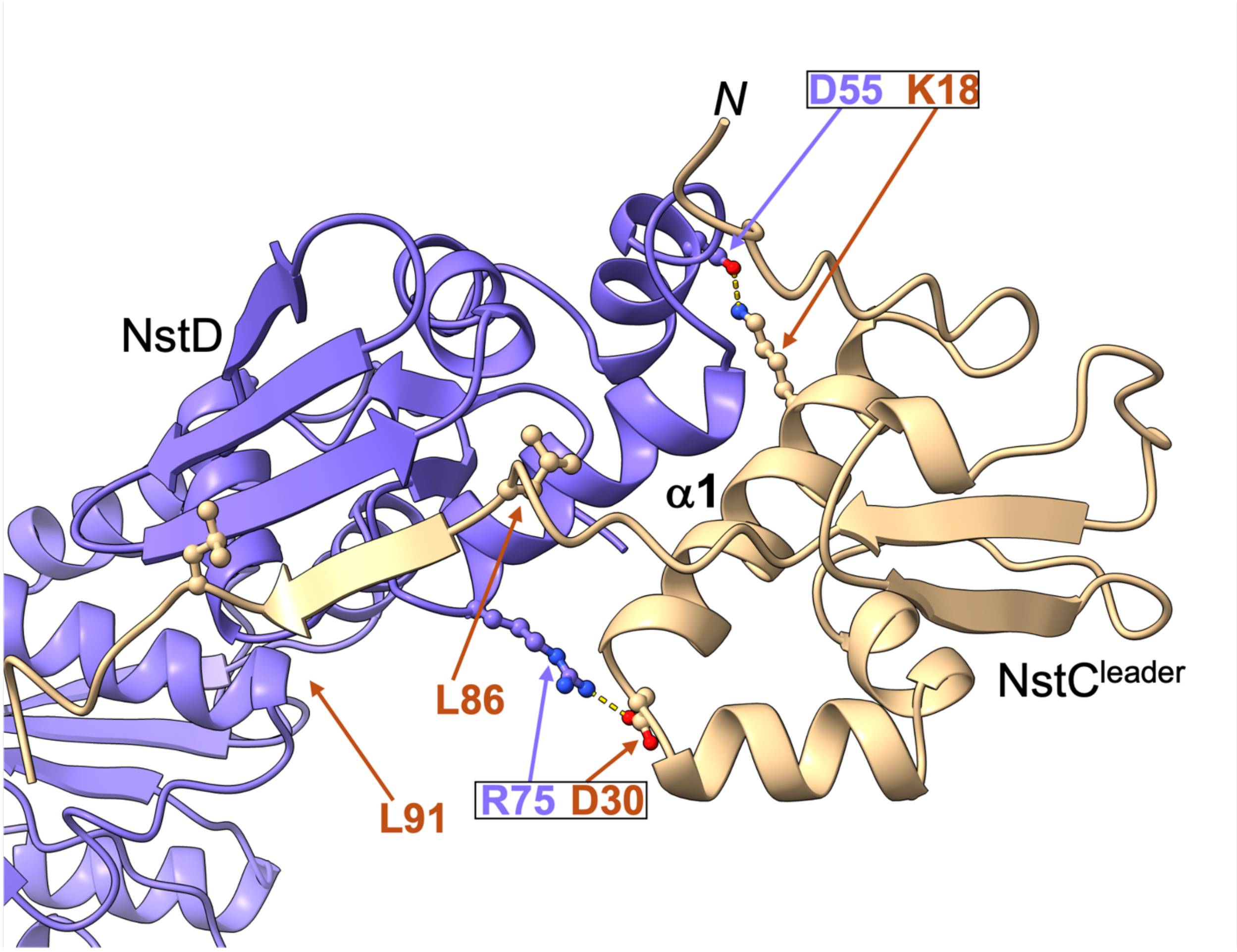

Figure S23: AlphaFold 3-predicted model of the NHLP NstC^{leader} (in brown) in complex with the YcaO NstD (in blue). For clarity, only one NstD monomer is illustrated. Note that Lys18 and Asp30 residues at either end of the NstC^{leader} α1 helix contact the YcaO RRE domain by making salt bridge interactions with the NstD RRE Asp55 and Arg75 side chains, respectively. Side chains of the two Leu residues—Leu86 and Leu91—that comprise the L(X)$_4$L motif in the NstC^{leader} are shown with the side chains in stick-ball representation and are placed proximal to a β-strand that extends the RRE domain's antiparallel β-sheet. The engagement of the NstC^{leader} α1 helix by polar interactions with the YcaO RRE domain is reminiscent of the MprE$_7^{leader}$/MprC interaction model.

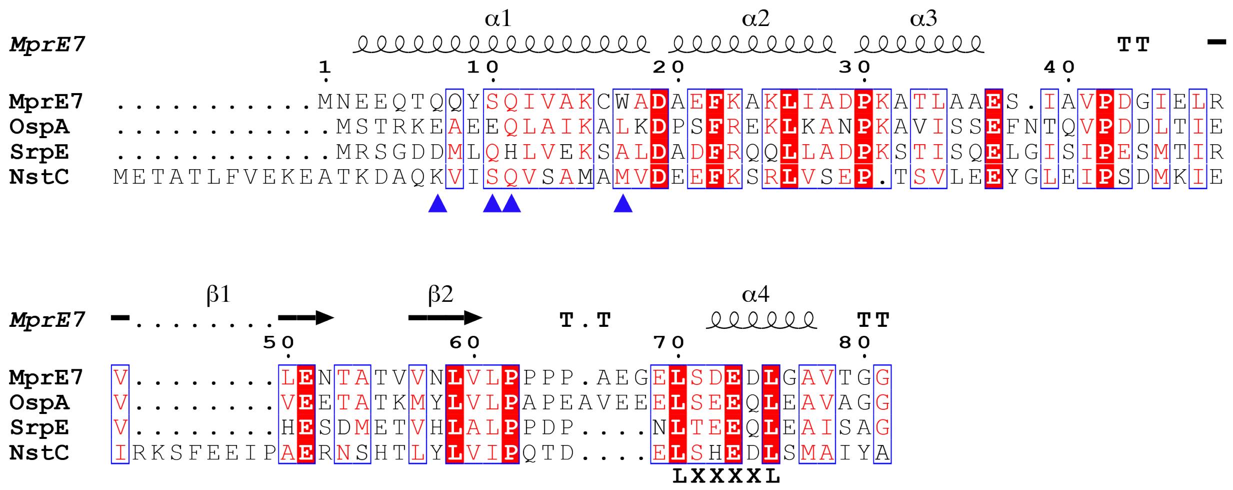

Figure S24: Sequence alignment between the NHLPs (from top): $\text{MprE}_7^{\text{leader}}$,[2] $\text{OspA}^{\text{leader}}$,[4] $\text{SrpE}^{\text{leader}}$,[5] and $\text{NstC}^{\text{leader}}$.[6] The secondary structural elements for the $\text{MprE}_7^{\text{leader}}$ (PDB: 8TB1) are superimposed upon the sequence alignment.[1] Note that the L(X)$_4$L motif is conserved in all four NHLPs. Despite the conservation of the L(X)$_4$L motif, the $\text{OspA}^{\text{leader}}$ and $\text{SrpE}^{\text{leader}}$ are unable to substitute the $\text{MprE}_7^{\text{leader}}$ to support MprC activity, likely due to the lack of conservation of residues that comprise the NHLP α1 helix. The four residues in the $\text{MprE}_7^{\text{leader}}$ α1 helix that were experimentally demonstrated in this study to be important in mediating the $\text{MprE}_7^{\text{leader}}$/MprC interaction are marked with blue triangles; note that these residues are not conserved in other NHLPs. Alignment was performed in Clustal Omega and analyzed in ESPript 3.

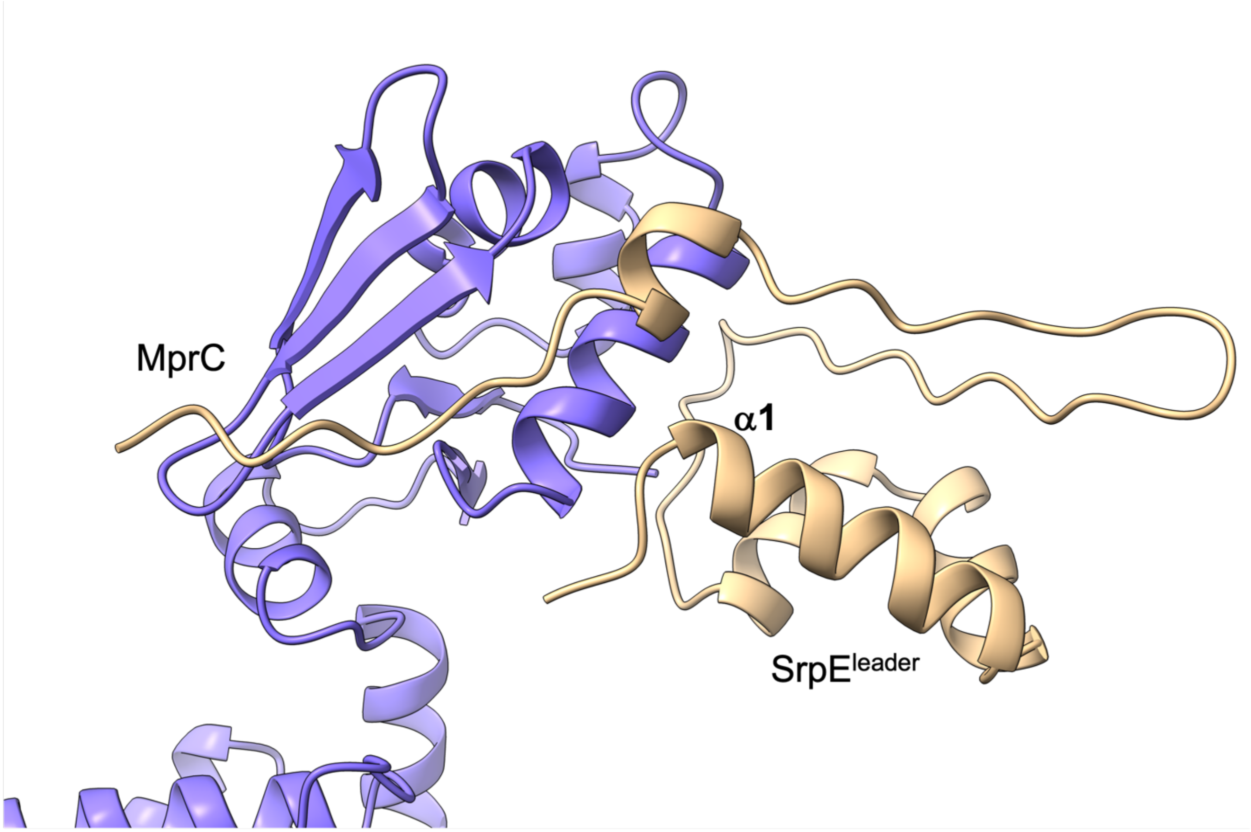

Figure S25: AlphaFold 3-predicted model SrpE^{leader}/MprC complex. For clarity, only one MprC monomer is shown. Note that the SrpE^{leader} α1 helix makes minimal contact with the RRE domain and the relative positioning of the NHLP trihelical nucleus to the RRE domain in the SrpE^{leader}/MprC complex is markedly different from that of the MprE$_7^{leader}$/MprC and the NstC^{leader}/NstD complexes.

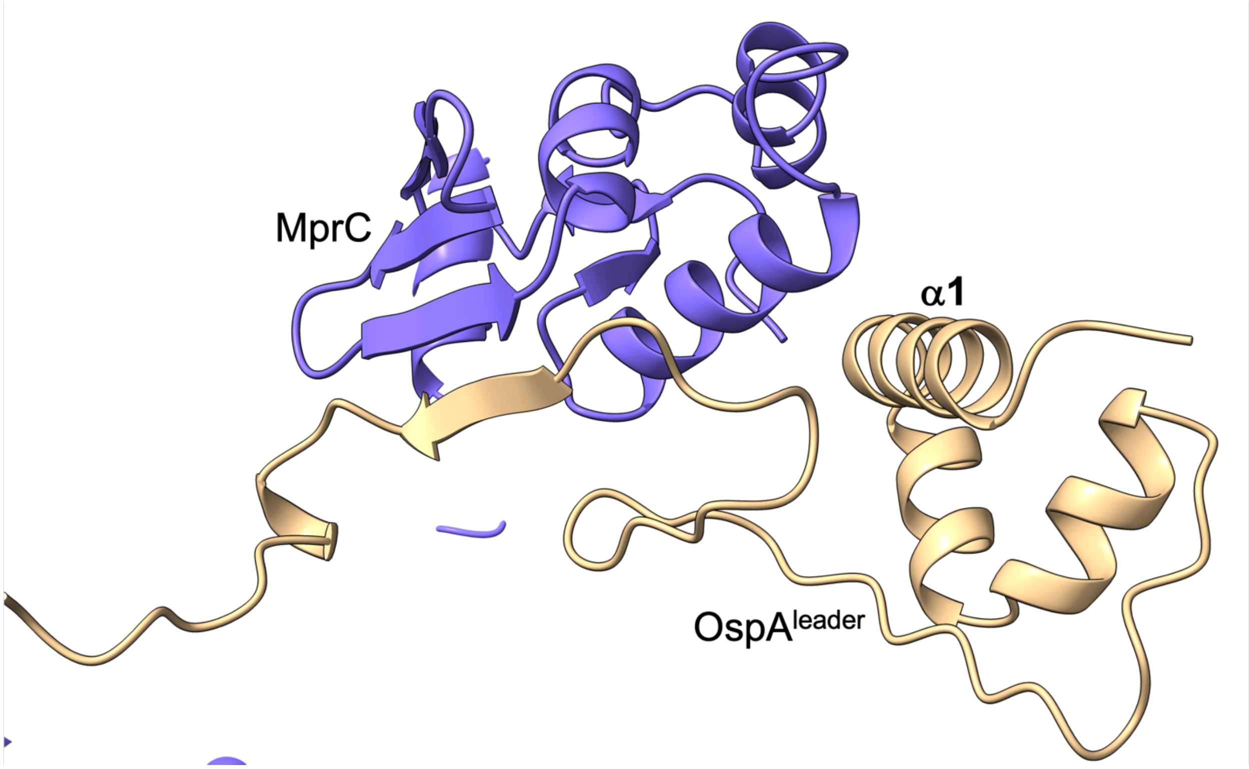

Figure S26: AlphaFold 3-predicted model OspA^{leader}/MprC complex. For clarity, only one MprC monomer is shown. Note that the OspA^{leader} α1 helix makes minimal contact with the RRE domain and the relative positioning of the NHLP trihelical nucleus to the RRE domain in the OspA^{leader}/MprC complex is markedly different from that of the MprE$_7^{leader}$/MprC and the NstC^{leader}/NstD complexes.

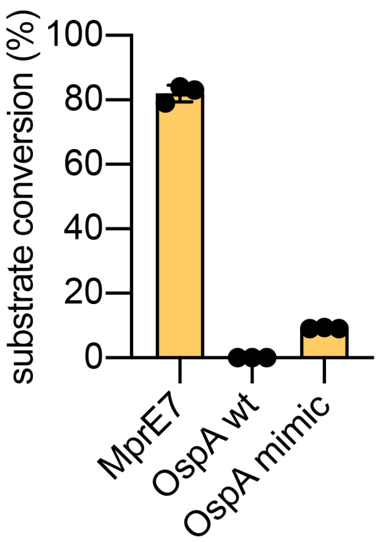

Figure S27: *In vivo* conversion of the GGPACAAK core when appended to the MprE7^{leader}, the OspA^{leader}, and the OspA$_{mimic}^{leader}$. Histograms represent mean ± standard deviations from three independent experiments. The substrate conversion of OspA$_{mimic}^{leader}$-GGPACAAK as compared to the MprE$_7^{leader}$-GGPACAAK is lower which denotes that further finessing of the NHLP leader will be required for future combinatorial production of RiPPs.

SUPPLEMENTARY REFERENCES

1. Nguyen, N. A.; Vidya, F. N. U.; Yennawar, N. H.; Wu, H.; McShan, A. C.; Agarwal, V., Disordered regions in proteusin peptides guide post-translational modification by a flavin-dependent RiPP brominase. *Nature Communications* **2024,** *15*, 1265.

2. Nguyen, N. A.; Cong, Y.; Hurrell, R. C.; Arias, N.; Garg, N.; Puri, A. W.; Schmidt, E. W.; Agarwal, V., A Silent Biosynthetic Gene Cluster from a Methanotrophic Bacterium Potentiates Discovery of a Substrate Promiscuous Proteusin Cyclodehydratase. *ACS Chemical Biology* **2022,** *17*, 1577-1585.

3. Bobeica, S. C.; Dong, S. H.; Huo, L.; Mazo, N.; McLaughlin, M. I.; Jimenez-Oses, G.; Nair, S. K.; van der Donk, W. A., Insights into AMS/PCAT transporters from biochemical and structural characterization of a double Glycine motif protease. *Elife* **2019,** *8*.

4. Bösch, N. M.; Borsa, M.; Greczmiel, U.; Morinaka, B. I.; Gugger, M.; Oxenius, A.; Vagstad, A. L.; Piel, J., Landornamides: antiviral ornithine-containing ribosomal peptides discovered through genome mining. *Angewandte Chemie International Edition* **2020,** *59*, 11763-11768.

5. Nguyen, N. A.; Lin, Z.; Mohanty, I.; Garg, N.; Schmidt, E. W.; Agarwal, V., An obligate peptidyl brominase underlies the discovery of highly distributed biosynthetic gene clusters in marine sponge microbiomes. *Journal of the American Chemical Society* **2021,** *143*, 10221-10231.

6. Delawská, K.; Hájek, J.; Voráčová, K.; Kuzma, M.; Mareš, J.; Vicková, K.; Kádek, A.; Tučková, D.; Gallob, F.; Divoká, P.; Moos, M.; Opekar, S.; Koch, L.; Saurav, K.; Sedlák, D.; Novák, P.; Urajová, P.; Dean, J.; Gažák, R.; Niedermeyer, T. J. H.; Kameník, Z.; Šimek, P.; Villunger, A.; Hrouzek, P., Discovery of nostatin A, an azole-containing proteusin with prominent cytostatic and pro-apoptotic activity. *Organic & Biomolecular Chemistry* **2025,** *23*, 449-460.
